# GSNOR provides plant tolerance to iron toxicity via preventing iron-dependent nitrosative and oxidative cytotoxicity

**DOI:** 10.1038/s41467-019-11892-5

**Published:** 2019-08-29

**Authors:** Baohai Li, Li Sun, Jianyan Huang, Christian Göschl, Weiming Shi, Joanne Chory, Wolfgang Busch

**Affiliations:** 1 College of Environmental and Resource Sciences, Zhejiang University, 866 Yuhangtang Road, Hangzhou, Zhejiang 310058 China; 2 Plant Biology Laboratory, Salk Institute for Biological Studies, 10010 N Torrey Pines Rd, La Jolla, CA 92037 USA; 3 Integrative Biology Laboratory, Salk Institute for Biological Studies, 10010 N Torrey Pines Rd, La Jolla, CA 92037 USA; 4 Gregor Mendel Institute (GMI), Austrian Academy of Sciences, Vienna Biocenter (VBC), Dr Bohr-Gasse 3, 1030 Vienna, Austria; 5 State Key Laboratory of Soil and Sustainable Agriculture, Institute of Soil Science, Chinese Academy of Sciences, No. 71 East Beijing Road, Nanjing, 210008 China; 6 Howard Hughes Medical Institute, Salk Institute for Biological Studies, La Jolla, CA 92037 USA

**Keywords:** Agricultural genetics, Genome-wide association studies, Natural variation in plants, Plant physiology

## Abstract

Iron (Fe) is essential for life, but in excess can cause oxidative cytotoxicity through the generation of Fe-catalyzed reactive oxygen species. It is yet unknown which genes and mechanisms can provide Fe-toxicity tolerance. Here, we identify *S-nitrosoglutathione-reductase* (*GSNOR*) variants underlying a major quantitative locus for root tolerance to Fe-toxicity in *Arabidopsis* using genome-wide association studies and allelic complementation. These variants act largely through transcript level regulation. We further show that the elevated nitric oxide is essential for Fe-dependent redox toxicity. GSNOR maintains root meristem activity and prevents cell death via inhibiting Fe-dependent nitrosative and oxidative cytotoxicity. *GSNOR* is also required for root tolerance to Fe-toxicity throughout higher plants such as legumes and monocots, which exposes an opportunity to address crop production under high-Fe conditions using natural GSNOR variants. Overall, this study shows that genetic or chemical modulation of the nitric oxide pathway can broadly modify Fe-toxicity tolerance.

## Abstract (web summary)

How plants deal with iron toxicity is still unclear. Here, the authors reveal that *S-nitrosoglutathione-reductase* (*GSNOR*) provides tolerance to iron toxicity by preventing iron-dependent nitrosative and oxidative cytotoxicity in Arabidopsis, legumes, and rice.

## Introduction

Iron (Fe) is an essential nutrient for every organism but can become a trigger to kill cells when present at elevated levels. In humans, Fe overload is correlated with many diseases such as those affecting vital organs like liver and heart, as well as neurodegenerative diseases including Alzheimer and early onset Parkinson^1^. In plants, serious symptoms of Fe toxicity have been recognized in rice for more than half a century^2^. In fact, Fe toxicity represents one of the most widely spread soil constraints for crop production in waterlogged soils, but also in a wide range of soil types including Ferralsols, Acrisols, Fluvisols, Podzols and Gleysols^3^, and can cause up to 10–90% of yield loss in rice^4,5^. The Fe concentration causing Fe toxicity in rice ranges widely, from 10 to >2000mgL^–1^ in the soil solution, and is highly dependent on other soil parameters such as geochemistry and nutrient levels, as well as on the particular rice variety^6^. It is estimated that 19% of the total area for rice production in Africa harbors a potential risk for Fe toxicity^7^. While engineering Fe toxicity tolerant rice varieties would be highly desirable, more than twenty attempts over the past two decades were not successful in identifying genes responsible for the variation of Fe toxicity tolerance in plants^8–13^.

The toxicity of Fe is so far mainly attributed to its reaction with hydrogen peroxide (H_2_O_2_) to generate the hydroxyl radical (OH˙) that is the most active reactive oxygen species (ROS)^14–16^. This reactive property of Fe can exert direct cellular damage via damaging biomolecules such as lipids or proteins, but also can regulate processes in growth and development. For instance, in humans, Fe-mediated ROS is linked with many diseases^1^, but also required to kill cancer stem cells through the ferroptosis (a specific type of cell death)^17,18^. In plants, Fe accumulation leads to primary root growth arrest induced by phosphorus deficiency and is associated with ROS over-generation^19^. While Fe dependent ROS production is seen as the major mechanism for Fe toxicity, studies in yeast suggest that Fe-catalyzed ROS production may not account for Fe toxicity^20^. Similarly, knockout of UPB1, a transcription factor controlling ROS homeostasis in the primary root of *Arabidopsis*^21^, does not affect the sensitivity of the primary root to high levels (500 µM) of Fe^22^. These results suggest that other components (additional to ROS) might be also required for Fe-mediated toxicity. On the other hand, ferritins (Fe binding proteins) seem to be the major components to buffer Fe overloading and consequently oxidative stress in both animals and plants^23,24^, but knockout of ferritin (triple mutant) does not change the sensitivity of the primary root in response to 500 µM Fe^22^, which suggests the primary mechanism for plant root tolerance to Fe toxicity is still elusive. For all the extensive research conducted in the area of Fe biology and Fe toxicity in plants and animals and despite its broad relevance, not only the major genes responsible for high Fe tolerance have yet to be identified in plants, but also the ROS-independent mechanisms of Fe toxicity are not well understood. Here, we identify S-nitrosoglutathione reductase (GSNOR) as a major genetic regulator required for Fe toxicity resistance throughout higher plants and show that the nitric oxide (NO) pathway can be key for modulating tolerance to Fe toxicity.

## Results

### GWAS identify a locus linked to Fe toxicity

**Fig. 1:**
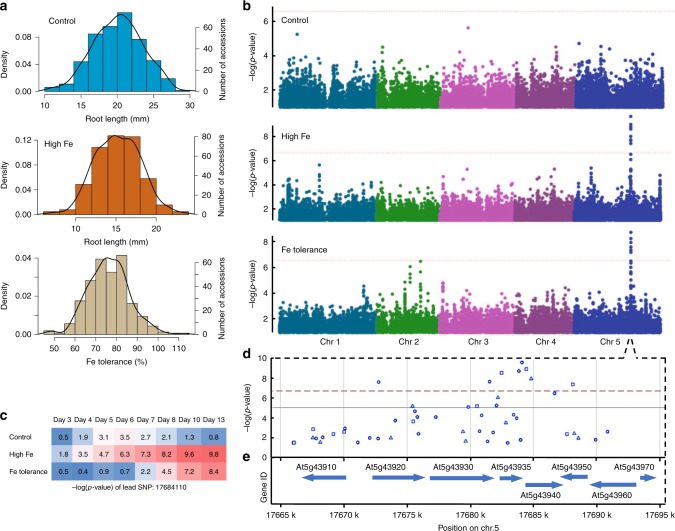
GWAS identifies GSNOR as associated with the root growth response to high Fe. **a** Distributions of root length and Fe tolerance index in 319 accessions at day 10. **b** Manhattan plots of GWAS using traits shown in **a**. Different colors indicate five chromosomes. The horizontal dashed lines indicated the thresholds of significance. **c** A heatmap of -log(*p*-value) of lead SNP identified at high Fe in **b** over the course of time. **d**, **e** Regional plots of the significant SNPs **d** and genes **e** identified at high Fe in **b**. GSNOR is encoded by At5g43940. Circle, triangle, and square denote the SNP to cause intergenic, non-synonymous_coding, and synonymous_coding changes, respectively. The source data of Fig. 1a are provided in a Source Data file

To identify genetic variants that confer plant tolerance to high Fe, we made use of natural variation of primary root growth responses in *Arabidopsis thaliana* to high Fe, as primary root growth is frequently used to evaluate heavy metal tolerance^25^ and also for high Fe toxicity in plants^22,26^. We measured primary root length at high Fe and Fe tolerance (root length of high Fe relative to the control) among 319 natural accessions from day 3 to day 13 after germination in control (1× MS) and high Fe (350 µM Fe) conditions (Fig. 1a; Supplementary Fig. 1a, b). The broad sense heritability of the observed variation ranged from 0.442 to 0.523 in control conditions and from 0.411 to 0.492 in high Fe conditions, with the highest value observed for day 10 (Supplementary Table 1). We then performed genome-wide association studies (GWAS) and found only one significant peak associated with root length of high Fe and Fe tolerance that exceeded the Bonferroni-corrected significance threshold of 5% (Fig. 1b, c; Supplementary Fig. 2a and b). This peak was located on chromosome 5 around the most significant single nucleotide polymorphism (SNP) at position 17684110 and was detected at multiple time-points (Fig. 1b, c; Supplementary Fig. 2b). Corroborating this peak, we also found this association using an alternative multi-trait GWAS approach (Supplementary Fig. 2c). As the SNP peak was the same regardless of using the absolute root length or relative root length at high Fe, or the multi-trait GWAS, we decided to use the absolute root length at high Fe for further analysis. Moreover, we chose to focus on the day 10 GWAS as the representative GWAS, as the broad heritability was highest at this day in control and high Fe conditions (Supplementary Table 1). We then analyzed the associated peak further and found eight significantly associated SNPs covering several genes (Fig. 1d, e). All of these SNPs were highly linked (Supplementary Fig. 3a). When conducting a conditional GWAS using the lead SNP:17684110, we found that only a single SNP still exceeded the Bonferroni-corrected threshold (Supplementary Fig. 3b). This genetic locus explained a notable proportion (20%) of the root growth variation at high Fe. The T-variant (54%) of this lead SNP was associated with higher Fe tolerance and the A-variant (46%) was associated with lower Fe tolerance within these 319 accessions (Supplementary Fig. 3c). Overall, this suggested that a genetic locus in that region is responsible for a major fraction of root growth variation among these 319 accessions upon high Fe.

### Natural allelic variation of *GSNOR* underlies Fe tolerance

To identify the causal gene for Fe tolerance within this genomic region, we first analyzed the gene expression of all ten genes surrounding the lead SNP in a publicly available data set (https://www.ebi.ac.uk/arrayexpress/experiments/E-GEOD-53197) containing both root and shoot expression data of 17 *Arabidopsis* accessions (Supplementary Fig. 3d, e). We reasoned that a causal gene might be differentially expressed already in control conditions in *Arabidopsis* accessions containing different lead SNP variants. Indeed, we found one gene, AT5G43940, which encodes a S-nitrosoglutathione reductase (GSNOR) and displayed a supporting expression pattern: it was significantly different between T-variant accessions (higher *GSNOR* expression) and A-variant accessions (lower *GSNOR* expression) in both roots and shoots under control conditions (Supplementary Fig. 3d, e). These results suggested that *GSNOR* might be the causal gene and expression variation of *GSNOR* might be responsible for the natural variation of root tolerance to high Fe.

**Fig. 2:**
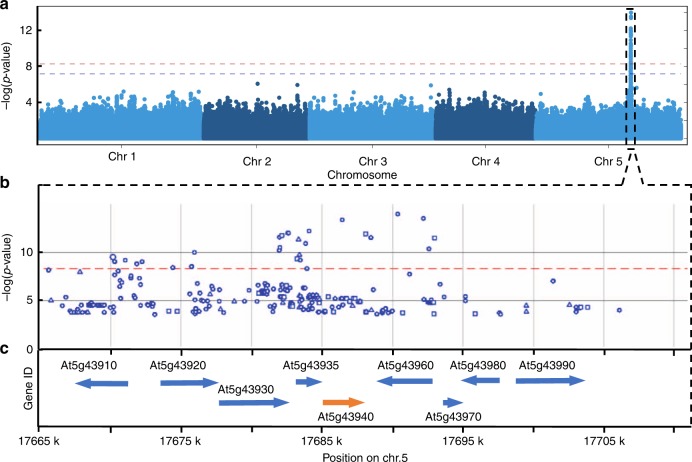
Natural GSNOR variants go along with transcription level variation. **a** Manhattan plots of *GSNOR* eQTL mapping of 665 *Arabidopsis* accessions using GWAS analysis. The expression levels of *GSNOR* gene are depicted in Supplementary Fig. 2f. **b**, **c** Regional plots of the significant SNPs **b** and gene models **c**. GSNOR is encoded by At5g43940. Circle, triangle, and square denote the SNP to cause intergenic, non-synonymous_coding, and synonymous_coding changes, respectively

To explore the pattern of natural variation of *GSNOR* transcript in a larger population, we analyzed the expression variation of *GSNOR* in shoots of 665 *Arabidopsis* accessions^27^ and found that its expression varied notably between different accessions (Supplementary Fig. 3f). We then performed an expression quantitative trait loci (eQTLs) mapping using GWAS with the *GSNOR* expression in these 665 accessions. Interestingly, we found a single significant SNP peak in immediate proximity to the *GSNOR* locus (Fig. 2a–c). These results suggested that the variation of *GSNOR* expression in the shoot can be caused by cis-regulatory variants. As there was a strong correlation between root and shoot expression of *GSNOR* of 17 accessions for which expression data was available (Pearson correlation coefficient is 0.599, *p*-value = 0.011), this hinted towards the relevance of cis-regulatory elements for the causality of the natural variation of root growth responses to high Fe.

**Fig. 3:**
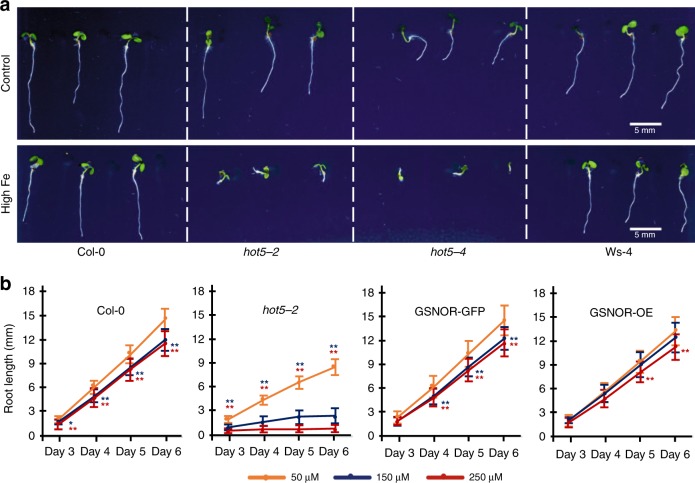
GSNOR is required for root tolerance to high Fe. **a** Seedlings of *gsnor* knockout mutants (*hot5-2* and *hot5-4*) and the respective wild-type (Col-0 and Ws-4) upon high Fe (250 µM) treatment for 6 days. Scale bar, 5 mm. **b** Mean root length of wild-type (Col-0), *gsnor* mutant (*hot5-2*), complemented line (*GSNOR-GFP*), and over-expression (*GSNOR-OE*) line. Error bars: standard deviation. *n* = 15 biologically independent samples. Asterisk * and ** represent the significant differences between high Fe (150 µM Fe in blue asterisk, 250 µM Fe in red asterisk) and the control as determined by Student’s *t*-test at *p*-value < 0.05 and 0.01, respectively. The source data of Fig. 3b are provided in a Source Data file

Consistent with our hypothesis that *GSNOR* is involved in Fe tolerance, root growth of both *gsnor* knockout mutants (*hot5-2* in Col-0 background and *hot5-4* in Ws-4 background)^28^ was very sensitive to high Fe (above 150 µM Fe) (Fig. 3a, b) and both mutant lines showed no root growth at 350 µM Fe in ½ x MS agar medium. The observed sensitivity to high Fe was not restricted to root growth but encompassed traits in the whole seedling as the leaves of *gsnor* knockout mutants were much smaller than the wild-type at 250 µM Fe (Fig. 3a). The roots of *hot5-2* mutants displayed 89% growth inhibition when Fe concentration increased from 50 µM to 250 µM, while only 20% inhibition was observed in wild-type roots at day 6 (Fig. 3b). 150 µM Fe already inhibited 70% of root growth in *hot5-2* mutants compared to the control, while it only caused 17% inhibition in wild-type at day 6 (Fig. 3b). Therefore, we considered the concentration of Fe at/above 150 µM in ½ x MS agar medium as high Fe in our subsequent experiments. Complementation of *hot5-2* using a *pGSNOR:GSNOR-GFP* transgene^29^ completely recovered the root growth of *hot5-2* at both 150 µM and 250 µM of Fe (Fig. 3b). Ubiquitous overexpression of *GSNOR*^30^ in Col-0 using the *35* *S* promoter (Supplementary Fig. 14) slightly enhanced tolerance to 150 µM Fe (Fig. 3b). Taken together, this demonstrated that *GSNOR* is required for root growth tolerance to high Fe, but that its ubiquitous overexpression in a tolerant accession does not necessarily lead to strong gains in Fe tolerance.

Next, we wanted to test whether natural *GSNOR* variants are causal for the variation of high Fe tolerance. We therefore conducted an analysis of the polymorphism patterns that distinguish Fe tolerant and sensitive accessions. When grouping accessions according to their Fe-sensitive A-variant and Fe-tolerant T-variant of the lead SNP and then analyzing their full genome sequences at the *GSNOR* locus, we found ample amount of sequence variation that distinguishes these two groups (Supplementary Fig. 4). The vast majority of this variation is non-coding and only one non-synonymous mutation in the amino acid sequence of *GSNOR* is strongly associated with the A-variant group of accessions (Supplementary Fig. 4). The complex pattern of polymorphisms within the A-variant group suggested that multiple alleles of *GSNOR* might contribute to the shorter roots in high Fe conditions. We therefore performed a haplotype analysis of the *GSNOR* sequence including the promoter region using the significant 8 SNPs identified by GWAS and found 10 haplotypes (at least present in 10 accessions) in the 319 accessions. Haplotype A, the biggest group, was found in 109 accessions and covers all variants of these 8 SNPs associated with high Fe sensitivity, while the second largest group, haplotype J, was found in 81 accessions and contains all variants of these 8 SNPs associated with high Fe tolerance (Supplementary Fig. 5). Based on root growth in high Fe, haplotype A is not significantly different from haplotype B and C, but is significantly different from the other haplotypes (Supplementary Fig. 5). These two biggest groups (haplotype A and J) defined by the haplotype analysis are essentially similar to the two groups defined by the two variants of the lead SNP. Therefore, we chose *GSNOR* from Col-0 (*GSNOR_Col-0*) in haplotype J to represent the Fe tolerant variant, while we chose *GSNOR* from Sf-2 (*GSNOR_Sf-2*) in haplotype A to represent the sensitive variant for further functional analysis.

**Fig. 4:**
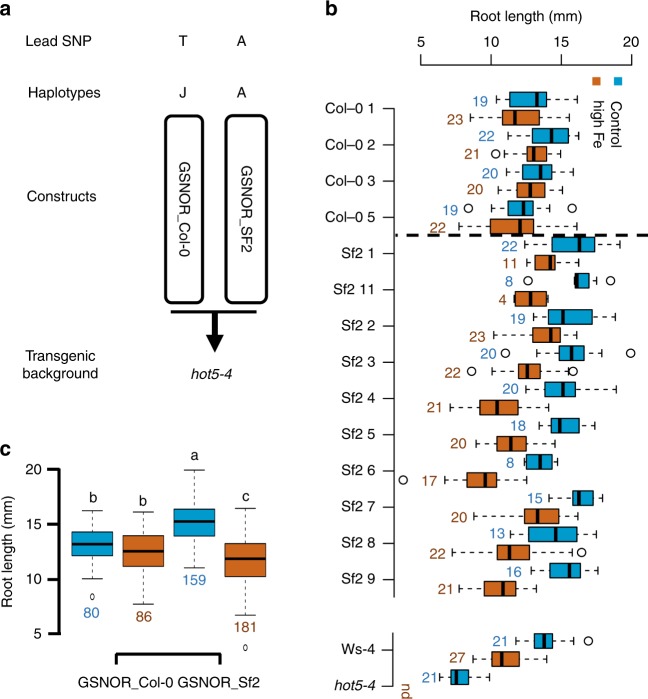
Natural *GSNOR* variants cause variation of root growth tolerance to high Fe. **a** Schematic of the constructs for *GSNOR* variants to generate transgenic lines. **b** Box plots for root length of T2 seedlings of ten *GSNOR_Sf-2* lines and four *GSNOR_Col-0* lines at day 6. *hot5-4* and *Ws-4* were included as the comparison for high Fe sensitivity. Not detected (nd) indicates no root growth of *hot5-4* was observed at 350 µM Fe conditions. **c** Box plots for the mean of ten *GSNOR_Sf-2* lines and four *GSNOR_Col-0* lines shown in **b**. Numbers below and next to boxes in **b, c** indicate the number (n) of T2 seedlings used for each line in the analysis. Different letters indicate significant difference according to Tukey’s HSD comparison (*p*-value < 0.05). The Source data of Fig. 4b are provided in a Source Data file

**Fig. 5:**
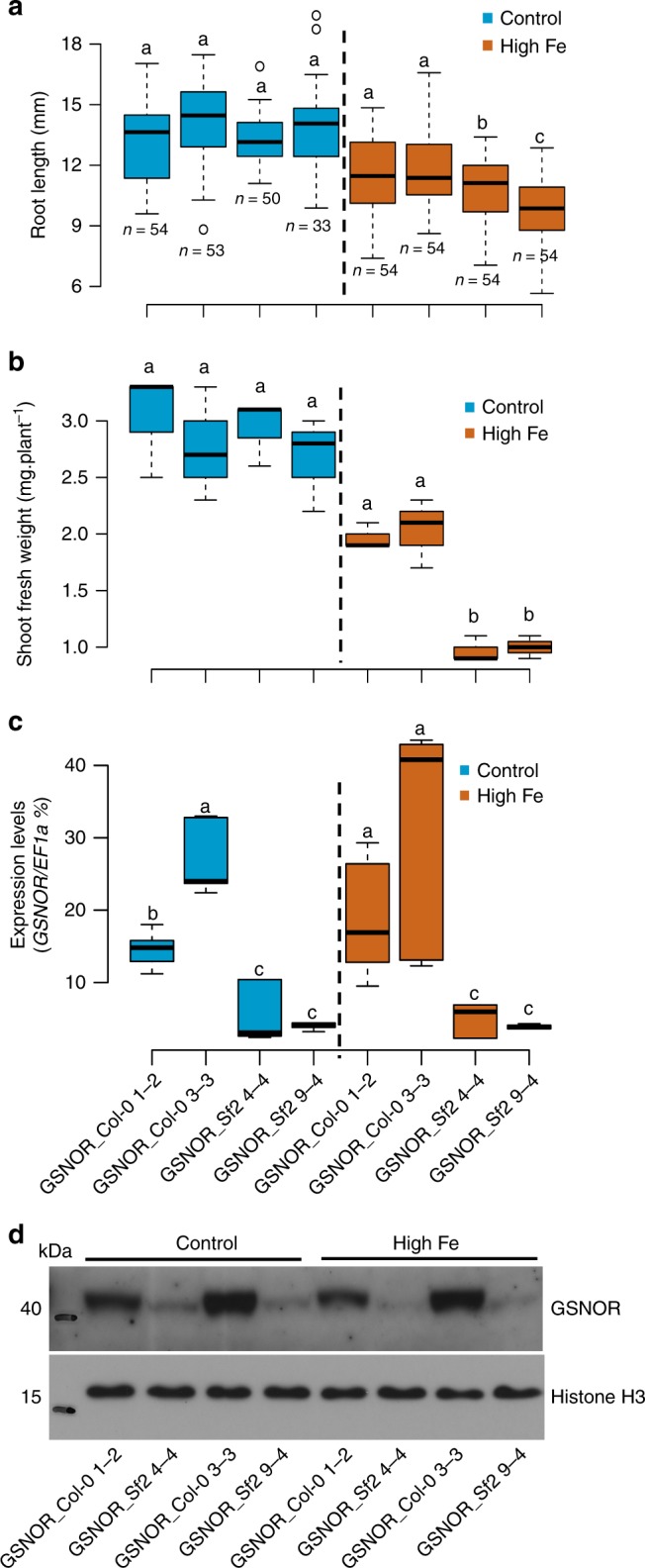
Natural *GSNOR* variants cause variation of GSNOR transcript levels and high Fe tolerance. **a** Box plots for root length of *GSNOR_Col-0* and *GSNOR_Sf-2* transgenic T3 lines at control and high Fe (350 µM Fe) at day 6. n, biologically independent samples. **b** Shoot biomass at day 15 (*n* = 3 biologically independent samples). **c** *GSNOR* expression in roots at day 6 (*n* = 6 including 3 biologically independent samples and 2 technical replicates for each sample). The seedlings were T3 generation. Different letters indicate significant difference with Tukey’s HSD comparison (*p*-value < 0.05) between different genotypes in a given condition. **d** Western blot analysis of GSNOR protein in 7-day-old seedlings at control and high Fe (350 µM Fe). Histone H3 protein is shown as the protein loading control. The source data are provided in a Source Data file

To experimentally test whether *GSNOR* variants of Col-0 (*GSNOR_Col-0*) and Sf-2 (*GSNOR_Sf-2*) lead to differences in Fe tolerance, we transformed these two *GSNOR* variants (encompassing 826 bp upstream of the translational start codon, the *GSNOR* gene and 166-bp downstream of the translational stop codon based on the Col-0 reference genome) into the *hot5-4* mutant (Fig. 4a), which has a better seed production than the *hot5-2* mutant. Consistent with the result of the haplotype analysis, the average root growth of all independent *GSNOR_Col-0* variant T2 lines showed significantly more tolerance to high Fe than all respective *GSNOR_Sf-2* variant lines (Fig. 4b, c). Similar differences were again observed in two T3 lines for each *GSNOR* variant (Fig. 5a). The high Fe root growth tolerance conferred by the *GSNOR_Col-0* alleles also correlated with an approximately two-fold increase in shoot biomass under high Fe compared to the sensitive allele that we measured in the T3 lines (Fig. 5b), demonstrating the relevance of *GSNOR* dependent high Fe tolerance at the organismal level.

In line with our observation of increased *GSNOR* transcript levels in Fe-tolerant accessions, the *GSNOR_Col-0* variant conferred a significantly higher *GSNOR* mRNA expression as well as protein level (Fig. 5c, d) and enzyme activity level (Supplementary Fig. 6c) than the *GSNOR_Sf-2* variant. Interestingly, the expression of *GSNOR* from both, a high Fe resistant variant (*GSNOR_Col-0*) and a high Fe sensitive variant (*GSNOR_Sf-2*), was not further induced by high Fe treatment (Fig. 5c), suggesting that the steady-state expression of GSNOR might be key for the increased Fe tolerance. Further support for the importance of *GSNOR* expression level for the observed effects of *GSNOR* natural variants came from the independently derived T3 lines harboring identical *GSNOR* sequences. Some of these independently derived *GSNOR_Sf-2* lines displayed different levels of *GSNOR* expression, presumably due to their T-DNA insertion site. As we measured not only expression level but also root growth in these lines, we could therefore analyze whether there is a relation between these two variables. Indeed, line 2, the most Fe tolerant line, displayed a significantly higher *GSNOR* transcript level than line 4 and line 9, which were significantly more sensitive to high Fe (Supplementary Fig. 6a, b).

Collectively, these results show that natural *GSNOR* variants cause variation of Fe toxicity tolerance and that this is most likely due to SNPs that cause *GSNOR* expression level variation.

### GSNOR counteracts Fe-dependent NO and H_2_O_2_ toxicity

As GSNOR activity confers Fe tolerance, we set out to explore the underlying molecular mechanism. GSNOR degrades S-nitrosoglutathione (GSNO) that is formed by the reaction of nitric oxide (NO) and glutathione (GSH)^31,32^ and thereby regulates NO signaling^28,33,34^ (Supplementary Fig. 7a). Previous studies had shown that both, NO and GSH are highly accumulated in the *GSNOR* knockout mutants^35^. We therefore first tested whether the accumulation of GSH arrests root growth in *gsnor* mutants at high Fe. For this, we analyzed the effect of GSH and L-Buthonine-sulfoximine (BSO, an inhibitor of gamma-glutamylcysteine synthetase, consequently reducing GSH synthesis) on the root growth response to high Fe. We found that root growth in *gsnor* mutants was decreased in high Fe when depleted of GSH (via treatment with BSO), but was not significantly changed by application of 0.5 mM GSH (Supplementary Fig. 7b). Overall, these results did not support a model in which internal GSH accumulation is the key factor to cause the highly Fe sensitive phenotype in the *gsnor* mutant.

**Fig. 6:**
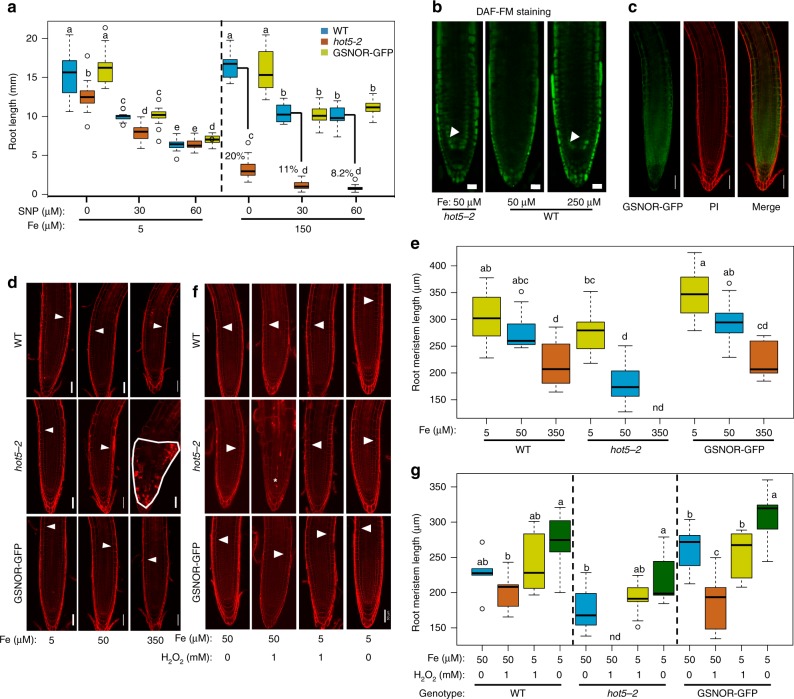
GSNOR alleviates Fe-dependent NO and H_2_O_2_ toxicities to promote root meristem growth. **a** Box plots for root growth upon high Fe and nitric oxide (NO) donor (sodium nitroprusside, SNP) at day 7 (*n* = 16 biologically independent samples). Root length of *hot5-2* relative to WT is shown in the right panel. **b** Fluorescence signal of root NO stained with DAF-FM at day 6. White arrows denote cortex cells in meristems. **c** Fluorescence signal of root expressing *pGSNOR:GSNOR-GFP* at day 6 under control conditions. **d** Root meristems stained by propidium iodide (PI) at day 6. **e** Box plots for root meristem of experiments depicted in (**d**) (*n* = 10 biologically independent samples). **f** Root meristems stained by PI upon H_2_O_2_ and Fe treatments at 7 days. White arrows indicate the border of elongation zone, while region outlined in white line and asterisk indicates cell death in the root meristem (**d**, **f**). **g** Box plots for root meristem length of experiments depicted in (**f**) (*n* = 9 biologically independent samples). Different letters indicate the significant differences according to Tukey’s HSD test (*p* < 0.05) between genotypes or different conditions in a given genotype. nd denotes not detected. Scale bar: 20 µm in (**b**); 50 µm in (**c**, **d** and **f**). The Source data of Fig. 6a, e, and g are provided in a Source Data file

We next tested the contribution of the NO accumulation to the highly Fe sensitive phenotype in *gsnor* mutants. Application of 30 µM or 60 µM sodium nitroprusside (a NO donor) significantly reduced root length in wild-type (37.4% and 39.2%), *gsnor* mutant (65.4% and 75.1%) and the complemented line (36.6% and 30.1%) at a level of 150 µM Fe, respectively (Fig. 6a). This showed that high levels of NO can cause *gsnor* mutants to be more sensitive to high Fe. We further tested the contribution of NO to Fe toxicity, by assessing root growth in another mutant *CAB Underexpressed 1*/ *Nitrous Oxide Overexpressor 1*, *cue1* that is known to accumulate high levels of NO in the root tip^36,37^. Again, root growth in the *cue1-6* mutant was more sensitive to high Fe compared to the wild-type (Supplementary Fig. 7c). Moreover, the application of cPTIO, a NO scavenger, significantly improved the root growth rate in *gsnor* mutants (2.9 fold) compared to wild-type (1.2 fold) at 150 µM Fe (Supplementary Fig. 7d). Consistent with the contribution of NO to Fe toxicity, we found that high Fe elevates NO levels in the root meristem, which mimics the phenotype of NO accumulation in *gsnor* mutants that display this elevated NO level already in control conditions (Fig. 6b). Consistent with the regulatory role in NO homeostasis, GSNOR is highly expressed in the root tip particularly in the meristem zone^29^ (Fig. 6c). Collectively, these results revealed that NO accumulation in *gsnor* mutants impairs root tolerance to high Fe and that genetical or chemical modulation of NO levels changes the tolerance of root growth to high Fe.

In contrast to the hyper-sensitivity at high Fe, root growth of the *gsnor* mutant was similar to the wild-type when grown on high NO but at a decreased Fe concentration of 5 µM (Fig. 6a). This suggested that the contribution of GSNOR in NO-mediated nitrosative inhibition of root growth depends on the Fe availability. We therefore hypothesized that Fe accumulation in the root tip may be one of the consequences of elevated internal NO level in the *gsnor* mutant, and thereby contributes to the reduced root growth rate. Consistent with this hypothesis, using Perl’s DAB staining, we found that Fe accumulation in the root tip of *gsnor* mutants was higher than the wild-type grown in control condition (50 µM Fe). However, this accumulation was not observed at lower Fe levels (10 µM Fe) in the medium (Supplementary Fig. 8a). This suggests that Fe accumulated in *gsnor* mutants largely depends on externally available Fe or apoplastic Fe. Consistently, reduction of Fe levels from the control to low Fe could further rescue the root growth of *gsnor* mutant (Fig. 6a; Supplementary Fig. 8b). Collectively, these data show that the protective role of GSNOR in nitrosative stress on root growth is highly correlated with Fe availability in the root tip.

The generation of Fe-dependent ROS is considered to be the major reason for Fe toxicity^14–16^. Therefore, GSNOR may be required for avoiding not only nitrosative stress but also oxidative stress. We therefore tested the root growth of *gsnor* mutant in response to exogenous H_2_O_2_, and found that root growth in *gsnor* mutant plants is very sensitive to H_2_O_2_ (Supplementary Fig. 8c and d). Moreover, *gsnor* hyper-sensitivity to H_2_O_2_ could be also alleviated by reducing Fe concentration in the medium (Supplementary Fig. 8c, d). Hence, similar to nitrosative stress, this result suggests that high Fe enhances H_2_O_2_ damage in *gsnor* mutants. Moreover, the protective role of GSNOR may be specific to Fe-dependent H_2_O_2_−mediated oxidative toxicity, as the *gsnor* shoots and roots were tolerant to paraquat^34^(Supplementary Fig. 8e), another common oxidative stress inducer and converting oxygen (O_2_) to the superoxide (O_2_^−^) radical^38^.

The root meristem is the center of cell proliferation and differentiation and thereby central for determining root growth^21^. We therefore examined the size of root meristem at different Fe levels and found that root meristem size in *gsnor* mutants was strongly decreased in high Fe conditions compared to wild-type (Fig. 6d, e). At 350 µM Fe in the medium, the root tips of *gsnor* mutants displayed cellular disorganization and radial swelling of cells in the outer tissue layers (Fig. 6d). At 50 µM Fe, *gsnor* mutant root meristems were still dramatically shorter than the wild-type (Fig. 6e) and only at 5 µM Fe, root meristem morphology and length recovered to that of wild-type plants (Fig. 6d, e). We also observed that high Fe caused a large number of dead cells in the *gsnor* root meristems using both propidium iodide (PI) and Sytox Orange staining (Fig. 6d; Supplementary Fig. 9). PI stains the walls of living plant cells but is also used as a marker for loss of membrane integrity and cell death, while Sytox Orange is a cell death marker^39^. Even at intermediate Fe levels (150 µM Fe) we observed frequent cell death in the root initials and their daughter cells in the meristem of *gsnor* mutant, but not in the wild-type (Supplementary Fig. 9). Moreover, we found that root meristem size, as well as cell death phenotypes in *gsnor* mutants were also very sensitive to H_2_O_2_, and depend on Fe availability (Fig. 6f, g). These results suggest that *GSNOR* determines the interaction of NO and Fe-dependent oxidative stress pathways to induce cell death in root meristems.

### GSNOR is required for high Fe tolerance in higher plants

**Fig. 7:**
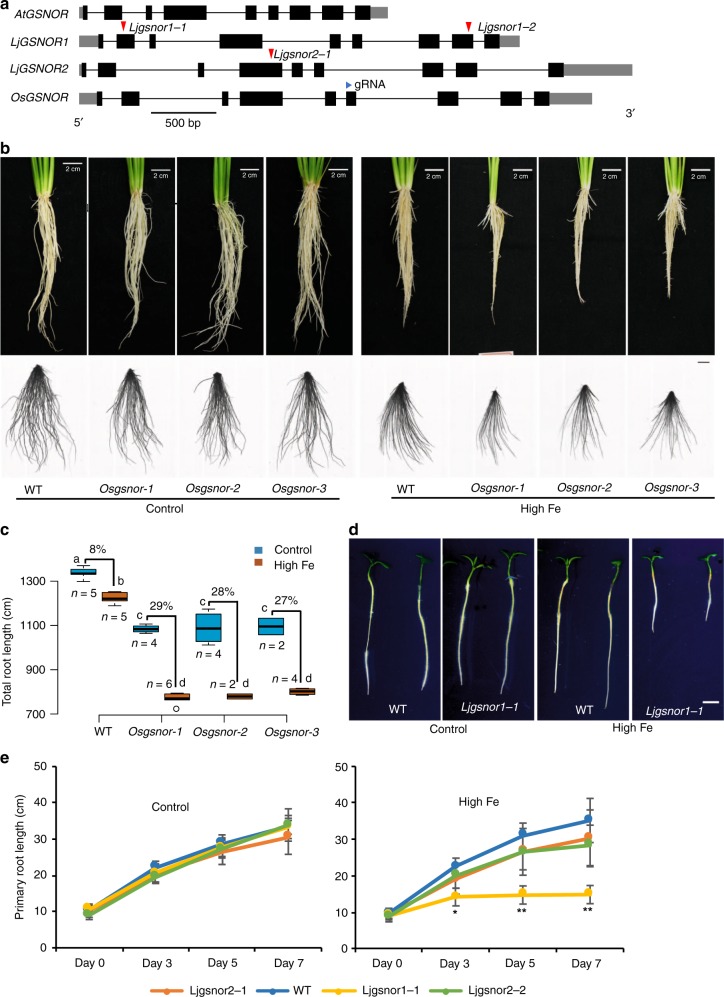
GSNOR function for Fe toxicity tolerance is conserved among higher plants. **a** Schematic representation of the gene structures and alleles of *GSNOR* from *Lotus japonicus* and rice. Red triangles indicate insert positions of LORE1 retrotransposon. Blue triangle indicates the position of guide RNA (gRNA) for CRISPR/Cas9. **b**, **c** Root growth of CRISPR/Cas9 knockout lines of *OsGSNOR* and WT. Upper panels, the roots before cutting; lower panels, the roots dispersed in water for total root length analysis. Scale bars: 2 cm. Quantified total root length is shown in box plots **c**. N, biological replicates. The percentage indicates the difference caused by high Fe. Different letters indicated the significant differences by one-way ANOVA with Tukey’s HSD test (*p* < 0.05). **d** Root growth at 50 µM Fe (control) and 350 µM Fe (high Fe) for 7 days. Scale bars: 5 mm. **e** Mean primary root length. Error bars: standard deviation. *n* = 8. Asterisk * and ** indicate the significant difference between WT and mutant lines at *p* < 0.05 and *p* < 0.01, respectively (Student’s *t*-test). The source data of Fig. 7c and e are provided in a Source Data file

The important function of *GSNOR* in conferring tolerance to high Fe in *Arabidopsis* prompted us to test whether it is conserved in other plant species. *GSNOR* is present as a single copy in the genomes of most plant species including the graminaceous species rice^29^ (Fig. 7a; Supplementary Fig. 10a). As high iron tolerance rice would be agronomically desirable in rice, we first chose to test the contribution of *OsGSNOR* in Fe toxicity tolerance in *japonica* rice (*Oryza sativa*) cultivar Zhonghua 11 (ZH11) by generating a knockout of *OsGSNOR* (Os02G57040) using the CRISPR/Cas9 system. Similar to *Arabidopsis*, three independent *OsGSNOR* knockout lines (*Osgsnor-1*, *Osgsnor-2*, and *Osgsnor-3*) containing frameshifts, displayed slightly reduced root growth under control conditions, but were dramatically more sensitive to high Fe (in hydroponics with 1000 µM Fe) compared to the wild-type ZH11 (Fig. 7b, c; Supplementary Fig. 11a, b). At this early stage, shoot growth was also slightly retarded in these three *OsGSNOR* knockout lines in control conditions, and much more so in high Fe conditions (Supplementary Fig. 11c). The reduction of height in *Osgsnor* knockout lines compared to wild-type amounted to 12% when treated with high Fe for 2 weeks. Thus, *OsGSNOR* contributes to both, root and plant tolerance to Fe toxicity in rice.

Duplications of the *GSNOR* gene can be found in some plant species, and commonly occur in legumes including *Lotus japonicus*, *Medicago truncatula* and *Glycine max* (Supplementary Fig. 10a). Phylogenetic analysis suggested both *LjGSNORs* from *Lotus japonicus* and *MtGSNORs Medicago trucatula* are most diverged from *AtGSNOR* (Supplementary Fig. 10b). This raised the question whether there is a subfunctionalization of *GSNOR* copies in species such as *Lotus japonicus* with *GSNOR* duplications. The gene structure and amino acid sequences of both *LjGSNOR1* (*Lj1g3v4528570*) and *LjGSNOR2* (*Lj0g3v0071329*) are still very conserved (89.2% and 90.1% of identity respectively, both of them are predicted to be alcohol dehydrogenase class-3-like clade) compared with *AtGSNOR* (Fig. 7a; Supplementary Fig. 10c). To test whether one or both homologs of *LjGSNOR* are involved in high Fe tolerance, we characterized *L. japonicus* knockout lines with LORE1 retrotransposon insertions^40^ in the exon of each of the two genes (Fig. 7a). We found that root growth of both *Ljgsnor1-1* and *Ljgsnor1-2* lines was more sensitive to high Fe, while this sensitivity was not observed in *Ljgsnor2-1* (Fig. 7d, e and Supplementary Fig. 11d). This increased root growth sensitivity was accompanied by a decreased size of the shoot system and increased accumulation of red pigments over time (Supplementary Fig. 11d). This showed that *LjGSNOR1* is required for high Fe tolerance in *Lotus japonicus* and suggests that *LjGSNOR2* has been subfunctionalized or is a homolog that has lost or is losing its function. A subfunctionalization is further supported by the absence of many phenotypes in *Ljgsnor1* mutants that are present in *Arabidopsis gsnor* mutants^28,29^ that include visible development defects at both young stages (shorter roots under the normal condition) and mature stages (shorter stem, increased branching, reduced fertility and shorter siliques) (Supplementary Fig. 11e, f), as well as by the distinct, moderately anticorrelated (r ~ 0.2) expression pattern of the two *LjGSNOR* genes (Supplementary Fig. 12).

## Discussion

As a major metal element in redox biology, Fe is required to induce radicals in many biological processes. However, this essential reactive property can lead to toxicity when Fe levels are too high. Our study identified *GSNOR* as underlying a major quantitative genetic locus controlling the natural variation of *Arabidopsis* tolerance to Fe toxicity. This is due to its central role in NO metabolism and subsequently in both nitrosative and oxidative stress regulation. Further, GSNOR is required for root tolerance to Fe toxicity not only in *Arabidopsis* but other dicots such as *Lotus japonicus*, and the monocot rice. Therefore, our findings not only shed light the underlying quantitative genetic and molecular mechanisms of plant adaptation to Fe-dependent toxicity, but also expose *GSNOR* as a prime target for genetic editing or marker-assisted breeding to develop crop varieties tolerant to Fe-dependent toxicity and help to increase rice productivity in regions with potential Fe toxicity particularly in West Africa. For this, additional studies need to be conducted in the relevant soils as Fe toxicity in plant not only depends on the Fe concentration in the soil, but is highly dependent on other soil parameters such as geochemistry and nutrient levels and on the particular genetic background.

While the amino acid sequence and the function of GSNOR for Fe toxicity tolerance is highly conserved at the interspecies level, we found a high diversity of regulatory SNPs in the promoter region and the introns of *GSNOR* within the *Arabidopsis* species. Complementation of the *gsnor* mutant with natural *GSNOR* variants indicated that polymorphisms that affect the expression of *GSNOR* contribute to the observed natural variation of root growth tolerance to high Fe. Consistent with the importance of the expression level for its function in tolerance to high Fe, modulating *GSNOR* expression levels in the mutant background was sufficient to increased Fe toxicity tolerance (Fig. 5; Supplementary Fig. 6). Interestingly, *GSNOR* expression is not induced by high Fe itself neither in *Arabidopsis* (Fig. 5c) nor in rice^41,42^, indicating that the base-line expression of *GSNOR* is important for its relevance for high Fe tolerance. The expression level effect is partly also supported by overexpression of *35* *S:GSNOR* in the Col-0 background (a high-Fe tolerant accession) that conferred a slightly more tolerance at 150 µM Fe. However, there was no tolerance difference at higher levels of Fe (Fig. 3b). As in this line, the *35* *S* promotor leads to a very high expression of GSNOR protein, as well as GSNOR activity^43^ (Supplementary Fig. 14), our results suggest that improved tolerance to Fe toxicity requires an optimal level of GSNOR (such as a level close to the GSNOR_Col-0 variant in the Ws-4 accession background) or a high GSNOR expression level in a specific cell type rather than in all cell types as conferred by the *35* *S* promoter.

While most of the data support the causality of GSNOR expression level variation for Fe tolerance, we cannot exclude the possibility that a nonsynonymous mutation (SNP:17684844) in GSNOR might also contribute to the phenotypic variation, even though it does not correlate with transcriptional variation of GSNOR. Nevertheless, high diversity (Supplementary Fig. 4) and strong linkage disequilibrium (Supplementary Fig. 3a, b) of the *GSNOR* related SNPs that we found make it difficult to distinguish the relative contribution of each SNP for the role of allelic variation for GSNOR function. It would be interesting to test the individual and combinatorial effects of these regulatory SNPs on *GSNOR* expression and on the binding affinity of upstream regulatory transcription factors.

Natural variants of *GSNOR* significantly contribute to Fe toxicity tolerance in *Arabidopsis* and it is likely to be caused by cis-regulatory variation. Rice also has large number of SNPs in the non-coding region of *GSNOR* (Supplementary Fig. 13). For example, we found 112 variations (primary allele frequency ranging 27% to 99.9%) including SNPs and INDELs between 2.0 kb upstream and 1.0 kb downstream of this gene from 4729 accessions using RiceVarMap 2.0 (http://ricevarmap.ncpgr.cn/v2/vars_in_gene/). Overall, these data suggest that *GSNOR* might be highly interesting for the development of Fe toxicity resilient crops. For this, it would be interesting to identify regulatory SNPs of rice *GSNOR* that could be directly used for gene editing and molecular marker-assisted selection for the breeding of Fe toxicity tolerance varieties. Similarly, non-coding SNPs of *GSNOR* were found to associate with the risk of childhood asthma in human^44^. These results suggest natural variation of GSNOR transcriptional levels may occur in both plants and animals.

Unlike the genes that confer hereditary diseases of Fe overload in humans and that are involved in Fe transport/homeostasis^1^, GSNOR seems to directly participate in the regulation of Fe-induced redox-dependent cytotoxicity. Fe-catalyzed ROS production is thought to be the major reason for Fe toxicity^14–16^. However, studies in yeast and plant indicate that Fe-catalyzed ROS production may not account for Fe toxicity^20,22^, which suggests that other components (additional to ROS) might be also required for Fe-mediated toxicity. We demonstrated that GSNOR protects root meristem growth and prevents cell death caused by Fe-dependent NO-induced nitrosative and H_2_O_2_-induced oxidative toxicity in *Arabidopsis*. These results revealed that NO is also required to generate Fe-dependent redox toxicity, which advances our understanding of the toxic mechanisms of Fe. Additionally, NO-mediated potassium homeostasis can also participate in the inhibition on the root growth by high Fe^45^. Thus, as the interplay of high Fe, ROS and reactive nitrogen species (RNS) is highly complex^46,47^, it will be very interesting to investigate these interactions and their impact of cytotoxicity.

Several reports indicate that NO promotes Fe uptake under Fe deficiency conditions^48,49^, while NO almost has no effect on either the expression of the Fe-acquisition genes or the ferric reductase activity at high level of Fe^50^. Since it has been reported that under control conditions three Fe deficiency responsive genes (bHLH100, 2.7-fold; bHLH39, 2.0-fold; bHLH38, 1.6-fold) were upregulated in the shoots of *gsnor* mutants compared to wild-type^29^, we explored the possibility that accumulation of NO in the roots of the *gsnor* mutants might increase Fe transport and accumulation in high Fe conditions. We therefore measured the root expression of *bHLH100*, *bHLH39*, *FIT1* all of which are key Fe deficiency induced transcription factors that directly activate the expression of ferric-chelate reductase FRO2 and the high-affinity ferrous Fe transporter IRT1 to increase Fe uptake and accumulation^51^. Expression of none of these genes was different between *gsnor* mutant and the wild-type in response to high Fe treatment (Supplementary Fig. 15). The same held true for expression of *FER1*, which encodes for the major ferritin protein that binds and stores Fe in plants (Supplementary Fig. 15). This strongly suggests that Fe uptake and transport are not among the major factors for the high Fe susceptibility of the *gsnor* mutant.

Our results also expose Fe as an important factor in this interplay between ROS and RNS, for instance in inducing cell death. This goes in line with the increasingly recognized role of Fe as important initiator of cell death in a variety of animal systems^17,18,52^. Considering the GSNOR sequence and enzyme activity in GSNO reduction, which is highly conserved among bacteria, plants, and animals including humans^28,29,31,32^, and the fundamental relevance of NO signaling, *GSNOR* and the NO pathway may be promising targets to tackle high-Fe-dependent disease conditions ranging from Fe-toxicity in plants and Fe-overload related conditions in humans.

## Methods

### Plant material and growth conditions

A total of 319 accessions of *Arabidopsis thaliana* were part of the Regional Mapping (RegMap) panel^53^. GSNOR knockout mutants *hot5-2* and *hot5-4* were in Col-0 and Ws-4 background, respectively^28^ (gifts from Dr. Elizabeth Vierling, University of Massachusetts, USA). The *proGSNOR:GSNOR-GFP* was in *hot5-2* background^29^. The GSNOR-overexpressing (GSNOR-OE) transgenic line^30^ and *cue1-6*^54^ (gift from Dr. Ulf-Ingo Flügge, University of Cologne, Germany) were in Col-0 background. The seeds of Ws-4 were from the Versailles Arabidopsis Stock Center. The *Lotus japonicus* mutants (Gifu background) were ordered from Lotus Base (https://lotus.au.dk/)^40^ and were genotyped with the primers listed in Supplementary Table 2. Plant ID of *Ljgsnor1-1*, *Ljgsnor1-2* and *Ljgsnor2-1* are 30033535, 30060068, and 30075087 in Lotus base, respectively.

*Arabidopsis thaliana* seeds were sterilized using chlorine gas produced from the mixture of 130 ml 10% sodium hypochlorite and 3.5 ml 37% hydrochloric acid in a sealed box for 1 h. Seeds that had been produced under uniform growth conditions were placed for 1 h in opened 1.5 ml in the sterilization box, and then were stratified in water at 4 °C for 3 days in dark. The seeds were sown on the 1% agar plates and then these plates were vertically positioned in racks in a growth chamber under a 16/8-h light-dark cycle at 21 °C. When we tried to grow these *hot5-2* and *hot5-4* mutants on 1× MS agar medium with 100 µM Fe, the plants did not develop any roots (also see the previous study^28^). We therefore used ½× MS agar medium with 50 µM Fe and found that the roots of *hot5-2* (which are slightly longer than *hot5-4*) reach around 60% of the wild-type length in this growth condition. We therefore used ½× MS agar medium from there-on for our further experiments except for the GWAS screening. If not specified differently, ½ x MS agar medium contained ½× MS salts, 1% (w/v) sucrose, 1% (w/v) agar (Duchefa Biochemie) and was pH5.7. Different concentrations of Fe(III)-EDTA or other chemical compounds as were added as indicated in the text.

Seed sterilization of *L. japonicus* was conducted according to the protocol^55^. The seeds were scraped using sand paper for 1–2 min, then sterilized with 0.5% sodium hypochlorite and 0.1% Triton-X solution for 18 min and rinsed with sterile water 5–6 times. Seeds were germinated on moist, sterile paper on vertical plates in a growth chamber under a 16/8-h light-dark cycle at 21 °C for 3 days, then transferred to the agar plates with different treatment as indicated.

Rice seeds were sterilized with 10% sodium hypochlorite for 10 min and rinsed 5 times with sterile water. The sterile seeds were soaked in water at 28 °C in dark for 3d, then were transferred to the modified Kimura B’ solution for another 2d under a cycle of 28 °C/16 h light and 25 °C /8 h dark, and with a light intensity of 400μmolm^−2^s^−1^ and 70% humidity. The modified Kimura B’ solution contained 0.5 mM MgSO_4_; 0.36 mM CaCl_2_; 0.25 mM KCl; 0.2 mM NaH_2_PO_4_; 50 μM H_3_BO_3_; 9 μM MnCl_2_; 0.3 μM CuSO_4_; 0.7 μM ZnSO_4_; 0.5 μM Na_2_MoO_4_, 100 μM Na_2_EDTA-Fe (II),7.5 mM NH_4_Cl,1 mM NaNO_3_, and at pH 5.5. For high Fe treatment, the seedlings were grown in the modified Kimura B’ solution with 1000 μM Na_2_EDTA-Fe (II) for another 2 weeks. The solution was exchanged every day.

### Root phenotyping

To examine the specific effect of high Fe on the root growth, natural variation of root length among 319 *Arabidopsis* accessions at control (1× MS with 100 µM Fe (III) -EDTA) and high Fe (1× MS with 350 µM Fe (III) -EDTA) conditions was conducted in parallel. Each plate contained eight accessions with three biological replicates. To account for positional effects within and between the plates, we plated 12 seeds for each accession over four plates in a permutated block design. Images of these plates were acquired with 8 CCD flatbed scanners (EPSON Perfection V600 Photo, Seiko Epson CO., Nagano, Japan) within half hour at the same time of at day3, day4, day5, day6, day7, day8, day10, and day13 after plating. Images were processed using the BRAT software^56^. Root length was measured using Fiji (http://fiji.sc/Fiji) in the experiments that included *hot5* mutants and those with *Lotus japonicus*. If not specified differently, for *Arabidopsis* root length measurement, days after plating were indicated in the text or the figure legends. In rice, the number of lateral roots (root length longer than 5 cm) was counted for each plant. For the total root length for each plant, the whole root system was cut at the junction between the shoot and the root and dispersed in water in a transparent tray. Images of these dispersed roots were acquired with a Dual Lens System scanner (Epson Perfection V700 PHOTO, China). The images were analyzed with WinRHIZO^TM^2012(Zeal Quest Scientific Technology Co., Ltd. China).

### Broad sense heritability calculation

All individuals of total root length in 319 natural accessions were used to calculate the broad-sense heritability (H_2_ = V_G_/V_P_), which is defined as the proportion of phenotypic variation (V_P_) due to genetic variation (V_G_) (estimated from the between-line phenotypic variance).

### Genome-wide association studies

Genome-wide association studies (GWAS) were performed for on the means of root length for each accession from 319 accessions at both the control and high Fe independently. In addition, GWAS was performed on the means of Fe tolerance index (%), which was the ratio of root length under high Fe and root length under the control conditions. GWAS was conducted using 250 K SNPs from the RegMap panel in a mixed model algorithm that has been shown to correct for population structure confounding using the AMM method on the GWA-portal (https://gwas.gmi.oeaw.ac.at/)^57^. SNPs with minor allele counts greater or equal to 15 were considered for the analysis. The significance threshold for SNP associations was set at 5% Bonferroni significance threshold to correct for multiple testing. A quantile-quantile (QQ) plot was generated and analyzed for each GWAS in GWA-portal. Linkage disequilibrium (LD) was visualized and analyzed using GWAPP (https://gwapp.gmi.oeaw.ac.at). Conditional (step-wise) GWAS^58^ was used to test whether SNPs associated independent of the primary associated SNP at a locus using GWAPP. With conditional GWAS analysis, the contribution of a locus for the observed phenotypic variation was also estimated using GWAPP. GWAS was performed in GWAPP/GWA-portal using default settings (no transformation and minor allele count (MAC) > 15). The multi-trait mixed model (MTMM), which considers the within-trait and between-trait variance components simultaneously for multiple traits^59,60^, was further used to generate multi-trait GWAS using the means of root length in control and high Fe conditions at day 10.

### Determining haplotypes

A single significant GWAS peak was identified with root length variation in 319 *Arabidopsis* at high Fe (Fig. 1b). This GWAS peak contained 8 significant SNPs. By counting the unique combinations of these 8 SNPs in 319 accessions, 10 groups of haplotypes were defined (frequency of each haplotype occurring in more than 10 accessions).

### Expression quantitative trait locus analysis

The data regarding the transcript levels of GSNOR in the shoots of 727 Arabidopsis accessions from 1001 genome project (https://www.ncbi.nlm.nih.gov/geo/query/acc.cgi?acc=GSE80744)^27^ was extracted. 665 out of these 727 accessions were available in 1001 G SNPs^61^, and were used to perform a GWAS-based expression quantitative trait locus (eQTL) analysis with 1001 G SNP data set in GWA-portal (https://gwas.gmi.oeaw.ac.at/) using the GSNOR transcript level as a trait.

### Construction of transgenic lines and CRISPR/Cas9 lines

For the comparison of *GSNOR* allele function, the 3115-bp genomic DNA sequence that encompassed 826 bp the upstream of the translational start codon, the *GSNOR* gene and 166-bp downstream of the translational stop codon based on the Col-0 reference genome, was amplified from Col-0 (haplotype J of T-variant in the lead SNP) and Sf-2 (haplotype A of A-variant in the lead SNP), respectively. These DNA fragments were introduced into a modified pGreen0229 vector at the EcoR I site. This modified pGreen0229 vector carried a *Pro35S:PM-mCherry* reporter gene, and kanamycin and basta resistance genes for bacteria and plants as a backbone, respectively^62^. The in-fusion cloning kit was used to construct all plasmids by following the user manual (Clontech, Japan). The fluorescence of mCherry reporter in the transformed seeds rather than antibiotic selection was used to select for positive transgenic lines using a fluorescence stereomicroscope (Leica MZ16 FA). Since the seed production is better in *hot5-4* compared to *hot5-2*, all plasmids were verified by sequencing and transformed into the *hot5-4* mutant (Ws-4 background) using agrobacterium-based floral dip method^63^. The cloning primers are listed in Supplementary Table 2.

All T1 seeds with mCherry signal were selected for further amplification. Based on the segregation of mCherry signals in seeds, all T2 lines (which derived from 4 (*GSNOR_Col-0*) or 10 (*GSNOR_Sf2*) independent T1 lines) rescued the root growth of *hot5-4* to the Ws-4 similar level or higher level in control and high Fe conditions (Fig. 4b). Two independent T2 lines with a single insertion that displayed a similar pattern of root growth responses to high Fe as the average root growth response of all T2 lines of the same genotype were chosen to represent each construct of *GSNOR_Col-0* and *GSNOR_Sf-2* variants at the T3 generation. These representative T2 lines were selected to amplify the T3 generation. These homozygous T3 plants were used to analyze the phenotype and GSNOR expression.

For CRISPR/Cas9 knockout lines of *OsGSNOR*, a single guide RNA (Supplementary Table 2) binding to the coding sequence of *OsGSNOR* (LOC_Os02g57040) was designed using the CRISPRdirect tool (http://crispr.dbcls.jp/)^64^. The sgRNA were introduced into the CRISPR/Cas9 binary vector pBGK032^65^. The *pBGK032-OsGSNOR* constructs were transformed into the calli of *japonica* rice cultivar Zhonghua 11 (ZH11) via *Agrobacterium tumefaciens* EHA105 using agrobacterium-mediated transformation^66^. The regenerated rice plants (T0) were selected by the hygromycin resistance and genotyped by sequencing using the primers (Supplementary Table 2). The biallelic, homozygous and heterozygous mutations in T0 plants were selected to amplify the seeds. The generation of *OsGSNOR* knockout lines was assisted by the Biogle Company (Hangzhou, China). The segregation of T1 seedlings were confirmed with genotyping and homozygous seedlings were used for phenotyping. Three independent homozygous lines with frameshifts: *Osgsnor-1*, *Osgsnor-2* and *Osgsnor-3* were chosen for phenotypic testing. They carry an insertion of ‘A’ or ‘T’ base at the position #654 of coding sequence and a deletion between #647 and #659 of the coding sequence downstream of the ATG start codon, respectively.

### Quantitative real time-PCR

Seedlings were grown on agar plates under control (½× MS with 50 µM Fe) or high Fe (½× MS with 350 µM Fe) conditions for 6 days after germination. Whole roots were collected and immediately frozen in liquid nitrogen. Twenty-five seedlings of each genotype were used as one biological replicate. Total RNA was extracted using the RNeasy Mini kit (QIAGEN GmbH, Hilden, Germany). First strand cDNA was synthesized using RevertAid Reverse Transcriptase (Themo Fisher Scientific, USA). 2× SensiMix SYBR & Fluorescein Kit (PEQLAB LLC, Wilmington, DE, USA) was used to prepare quantitative real time-PCR (qRT-PCR) reactions and PCR was performed in a Roche Lightcycler 96 (Roche) instrument. Three biological replicates and two technical replicates were analyzed for each gene. Relative quantifications were performed for all genes with the gene *elongation factor 1 alpha* (*EF1a*, AT5G60390) used as an internal reference. The primers used for qRT-PCR are shown in Supplementary Table 2.

### Histochemical Fe staining with Perls/DAB stain

Fe accumulation in roots was assayed with Perls/DAB staining^67^. Briefly, 6-day-old seedlings were washed with 2 mM CaSO_4_ and 10 mM EDTA for 30 min and rinsed in water 5 min, then incubated in 1 mL of 2%(v/v) HCl and 2%(w/v) K_4_[Fe(CN)_6_] for 30 min. These samples were then washed 3 times with water and incubated in 1 mL of 0.01 M NaN_3_ and 0.3% H_2_O_2_ in methanol. After 30 min, the samples were washed 3 times with 1 mL 0.1 M KPO_4_ buffer in pH 7.4. Then, the samples were incubated in 1 mL of 3,3′-Diaminobenzidine tetrahydrochloride hydrate (DAB) solution (0.025% DAB, 0.005% H_2_O_2_ and 0.005% CoCl_2_ in 0.1 M KPO_4_ in pH 7.4) in the dark. After 10 min, the samples were washed at least 3 times with water. Finally, the Fe staining in roots was observed with an optical microscope.

### Fresh shoot biomass measurement

Shoot tissues from four seedlings that grown on a single plate were collected as a biological replicate (one pool). Three biological replicates for each condition were analyzed. The shoot fresh weight per plant was calculated by dividing the weight of the pool by the number of plants.

### GSNOR activity assay

GSNOR activity was measured by monitoring the decomposition of NADH at 340 nm^32^. Approximately 30 seedlings (8-day-old) were collected and weighted. The samples were ground and extracted with 4 volume (4 μlmg^−1^) of 50 mM HEPES buffer ((20% glycerol, 10 mM MgCl_2_, 1 mM EDTA, 1 mM EGTA, 1 mM benzamidine, and 1 mM ɛ-aminocaproic acid, pH 8.0), centrifuged to remove insoluble material at 4 °C, 16,000 *g* for 15 min. The supernatants were further clarified using Zeba™ Spin Desalting Columns (Thermo-Fischer). The desalted fraction (10 μL) was added to the 190 μl assay mix [20 mM TRIS-HCl (pH 8), 0.2 mM NADH, and 0.5 mM EDTA] with 400 μM GSNO or H_2_O, respectively. The NADH decomposition was monitored for 10 min after the addition of GSNO using a Tecan Safire II multi-mode plate reader. The rates were corrected for background NADH decomposition of each extract containing no GSNO. Another desalted fraction (10 μL) was used for determining the total protein concentration by using Pierce™ Rapid Gold BCA Protein Assay Kit (Thermo-Fischer) with this Tecan Safire II multi-mode plate reader. The corrected reduction rate of NADH was normalized against total protein amount. At least three biological replicates for each experiment were performed, the results of two independent experiments were similar, and the result of one independent experiment was presented in the figure.

### Immunoblotting

Seven-day-old seedlings (around 30 plants) grown on ½ MS medium or ½ MS medium supplemented with 300 µM Fe were harvested, weighed and ground in liquid nitrogen to fine powder. In total 5 volumes (5 µl. mg^−1^) of 2 × NuPAGE lithium dodecyl sulfate (LDS) sample buffer (Thermo-Fischer) with Tris(2-carboxyethyl) phosphine Hydrochloride (TCEP) was added to the samples and then boiled for 5 min. After centrifugation at 15,000 × *g* at 4 °C for 15 min, 15 μL of supernatant were loaded, separated in a NuPAGE 4–12% Bis-Tris protein gel (Thermo-Fischer), followed by a wet transfer to nitrocellulose membrane (Bio-Rad). The GSNOR protein was detected by probing the membrane with anti-GSNOR antibody (AS09 647, Agrisera) at a dilution of 1:2000. Loading was controlled by using a rabbit anti-histone H3 antibody (9715 S, Cell Signaling Technology) at a dilution of 1:2000. Signal was detected using the SuperSignal™ West Pico PLUS Chemiluminescent Substrate (Thermo-Fischer). At least two independent biological replicates (the samples from two different experiments) were performed for the western blot. The representative images from one experiment were presented.

### Measurements of meristem size and cell length in roots

Six-day old *Arabidopsis* seedlings were stained with 15 µM PI staining for 10 min and rinsed with water. The profile of root structure was visualized with PI staining and an LSM 700 confocal microscopy (Zeiss). The meristem size was measured from the quiescent center cells to first elongated cortex cell (median optical section was more than twice in length than in width). The cell length was measured from 30 fully elongated cortex cells in approximate 10 individual roots for each genotype in a given condition.

### Nitric oxide staining of roots

6-day old seedlings were incubated in 5 µM 3-amino, 4-aminomethyl-2,7-difluorofluorescein diacetate (DAF-FM DA, Molecular Probes, USA) in 10 mM Tris–HCl with pH 7.5 for 40 min at 25 °C in the dark, and then were washed three times with fresh buffer (10 mM Tris–HCl, pH 7.5) for 15 min. The fluorescence was observed with an LSM 700 confocal microscope.

### Cell death staining

Cell death in the root tip was stained with 15 µM PI (Sigma) or 250 nM Sytox Orange nucleic acid Stain (Molecular Probes, Invitrogen) for 10 min, and rinsed twice with water before imaging with an LSM 700 confocal microscopy^39^.

### Microscopy

All fluorescence was observed using an LSM 700 confocal microscopy (Zeiss). The excitation and emission wavelengths were set as follows: 488 nm and 485–545 nm for DAF-AM staining and GFP; 555 nm and 570–670 nm for PI and Sytox Orange staining. The images were analyzed with Fiji (http://fiji.sc/Fiji). For microscopy observations, at least 8 individual roots were analyzed for each genotype in a given condition in each independent experiment. At least 2 independent experiments were performed. The representative images from one experiment were presented.

### Quantification and statistical analysis

All values are shown with box plots or as the means ± standard deviation (SD) and the number (n) of samples is indicated in figures or figure legends. For box plots, the midline is the median of data; the upper and lower limits of the box are the third and first quartile, respectively; the whiskers are 1.5 times the interquartile range from the top (bottom) of the box to the furthest datum within that distance. No statistical methods were used to pre-determine sample size. Significant differences between two samples for time-course experiments were determined with Student’s *t*-test. Significant differences for multiple comparisons for single point experiment were determined by one-way or two-way ANOVA with Tukey’s HSD test as indicated in figure legends. Statistical analyses and analysis of broad sense heritability were performed in the RStudio software (https://www.rstudio.com/).

### Reporting Summary

Further information on research design is available in the Nature Research Reporting Summary linked to this article.

## Supplementary information

Supplementary Information

Peer Review

Reporting Summary

Source Data

## Data Availability

Data supporting the findings of this work are available within the paper and its Supplementary Information files. A reporting summary for this Article is available as a Supplementary Information file. The datasets generated and analyzed during the current study are available from the corresponding authors upon request. The source data underlying Figs. 1a, 3b, 4b, 5a–d, 6a, e, g, 7c, e, and Supplementary Figs. 1a, 3c-f, 5, 6a–c, 7b–d, 8d, 11b, c, 12, 14, and 15, as well as Supplementary Table 1 are provided as a Source Data file.
