# Supplementary material for: GSNOR provides plant tolerance to iron toxicity via preventing iron-dependent nitrosative and oxidative cytotoxicity
Source: Nat Commun. 2019 Aug 29;10:3896. doi: 10.1038/s41467-019-11892-5 (PMC6715714; doi:10.1038/s41467-019-11892-5)
Supplement: Supplementary file 1 — Supplementary Information [file 41467_2019_11892_MOESM1_ESM.pdf]

GSNOR provides plant tolerance to iron toxicity via preventing iron-dependent nitrosative and oxidative cytotoxicity

Li *et al.*

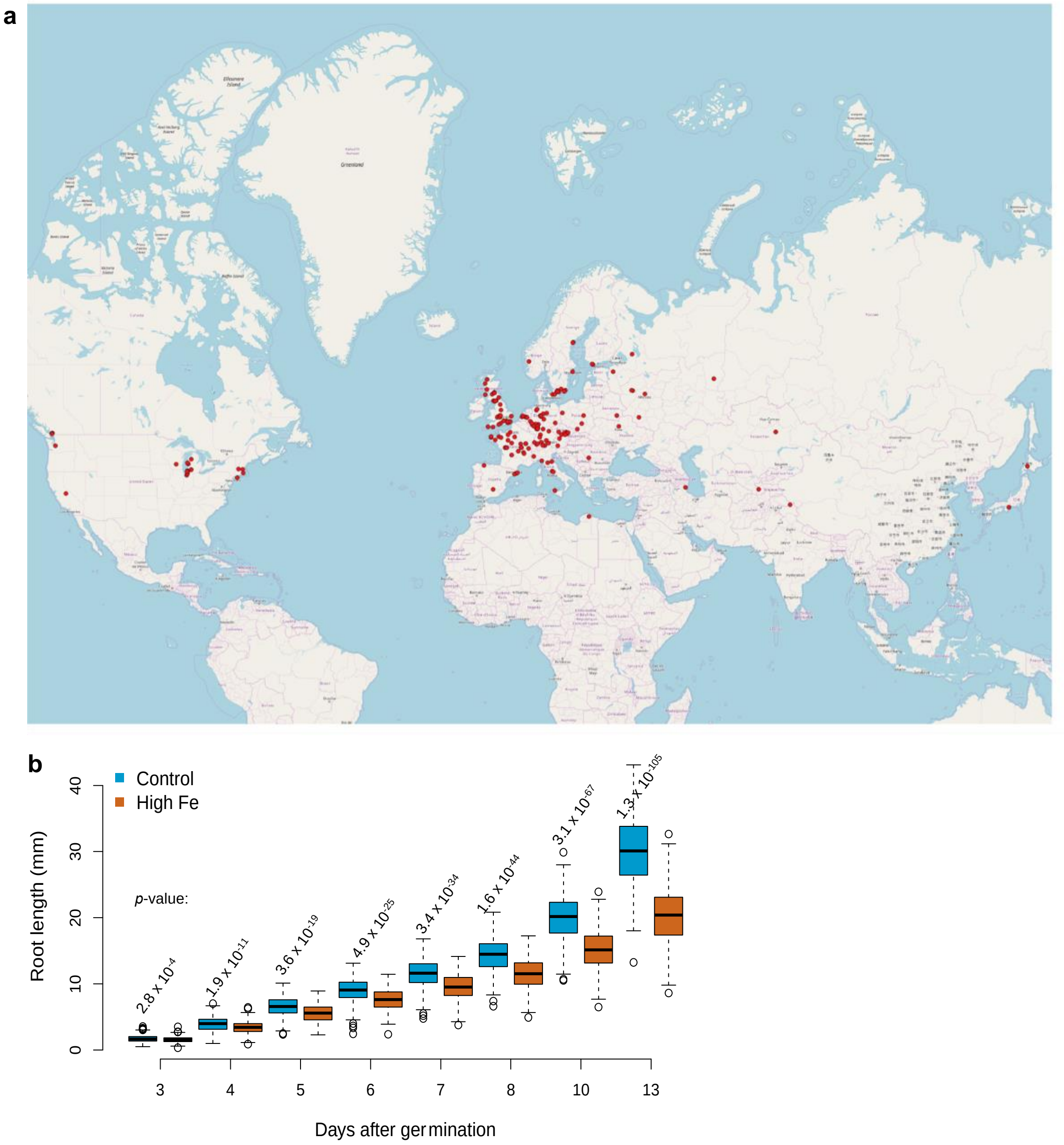

Supplementary Figure 1. Natural variation of root growth responses to high Fe in *Arabidopsis*. a, Geographic distribution of 319 accessions of *Arabidopsis thaliana* used in this study. Map is plotted using data available under the Open Database License © OpenStreetMap (http://www.openstreetmap.org/copyright). **b**, Box plots for the primary root length of 319 *Arabidopsis* accessions under control and high Fe (350µM) conditions. *P*-value of Student's *t*-test for comparing growth in control and high Fe for each time point is indicated in figure. The source data of Supplementary Figure 1a are provided in a Source Data file**.**

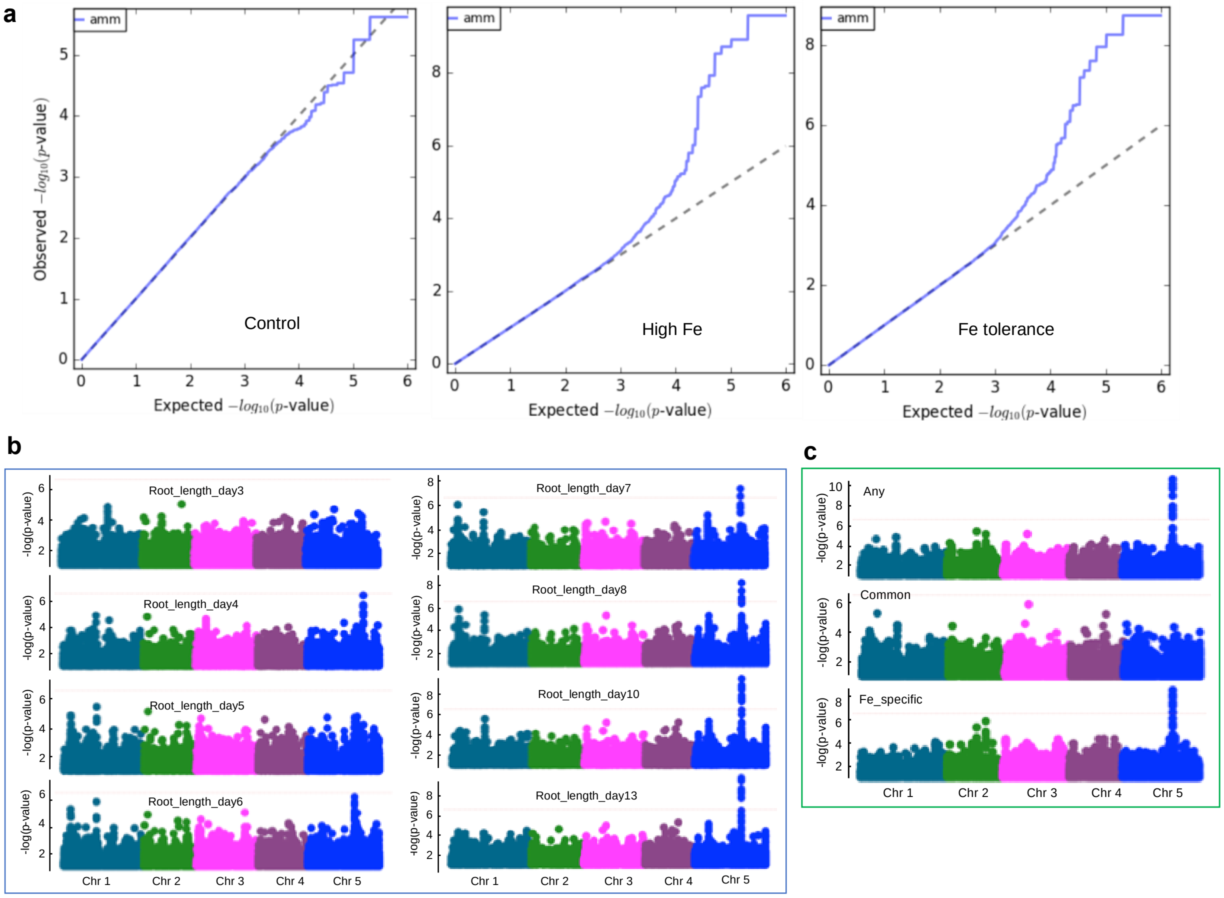

Supplementary Figure 2. Genome-wide association studies of root growth responses to high Fe in *Arabidopsis*. a, Quantile-quantile (QQ) plots of GWAS analysis in Fig. 1B. **b**, Manhattan plots of GWAS analyses using the primary root length in high Fe from day3 to day13 after germination. **c,** Manhattan plots for multi-trait GWAS analysis for high-Fe dependent primary root length at day10.

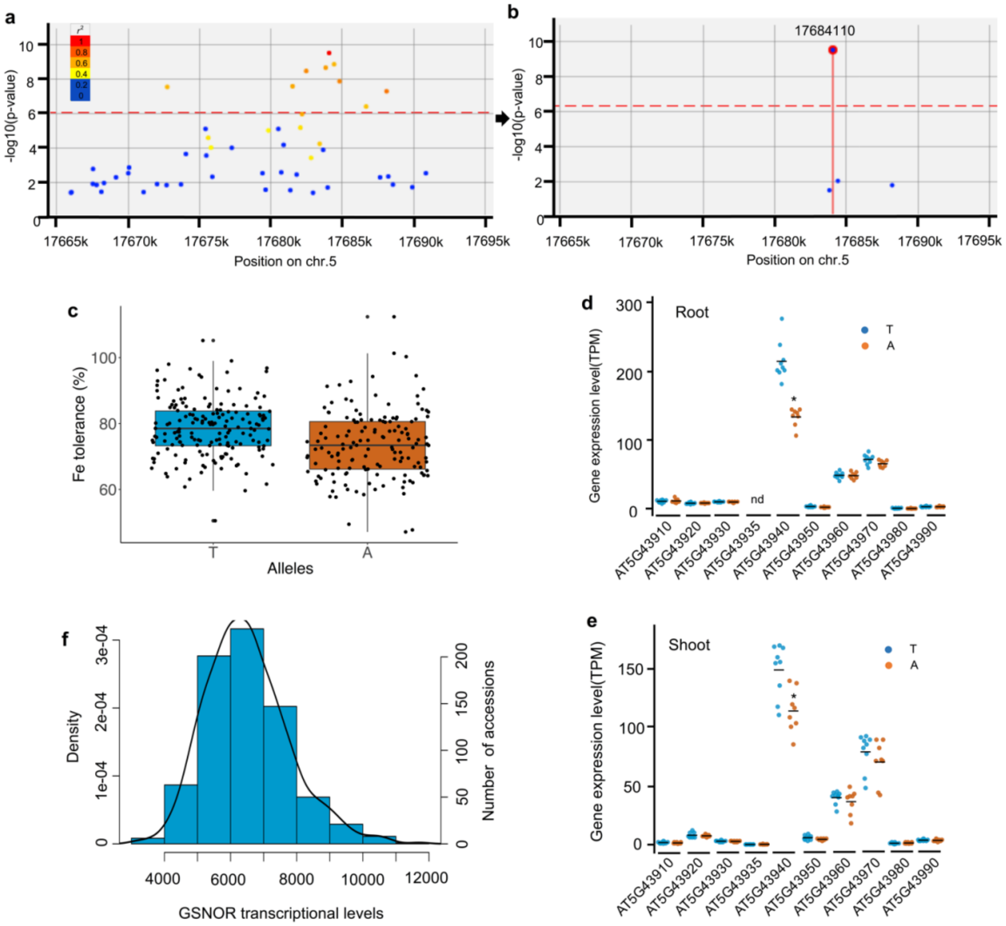

Supplementary Figure 3. Analysis of top SNPs identified by GWAS for root length in high Fe. a, Linkage Disequilibrium (LD) analysis of the lead SNP identified by GWAS at high Fe in Fig. 1B. GWAS -log(10)(p-value) of SNPs shown on y axis, while chromosome position of SNPs shown on x axis. The square of the correlation coefficient (r^2) between different SNPs are shown in different colors. **b**, Conditional GWAS analysis with the lead SNP indicated in (**a**). The position of lead SNP shown in figure. **c**, Box plot of Fe tolerance at day10 grouped by the two variants of the top SNP. Each dot shows a phenotype value of an accession. n = 171 and 148 accessions for allele T and A, respectively. **d**, **e,** Dot plots and the mean of expression levels of the ten genes surrounding the lead SNP in roots (**d**) and shoots (**e**) of 17 *Arabidopsis* accessions grouped by the two variants of the lead SNP (resistant allele T (n = 9 accessions) and sensitive allele A (n = 8 accessions)). Asterisk (*) indicates the significant difference by Student's *t*-test *at p*-value < 0.05. Data from (https://www.ebi.ac.uk/arrayexpress/experiments/E-GEOD-53197). **f**, Histogram and density curve of GSNOR transcript level in the shoots of 665 *Arabidopsis* accessions. Data was derived from 1001 *Arabidopsis* genomes transcriptome datasets[27]. GSNOR is encoded by At5g43940. The source data of Supplementary Figure 3c-f are provided as a Source Data file.

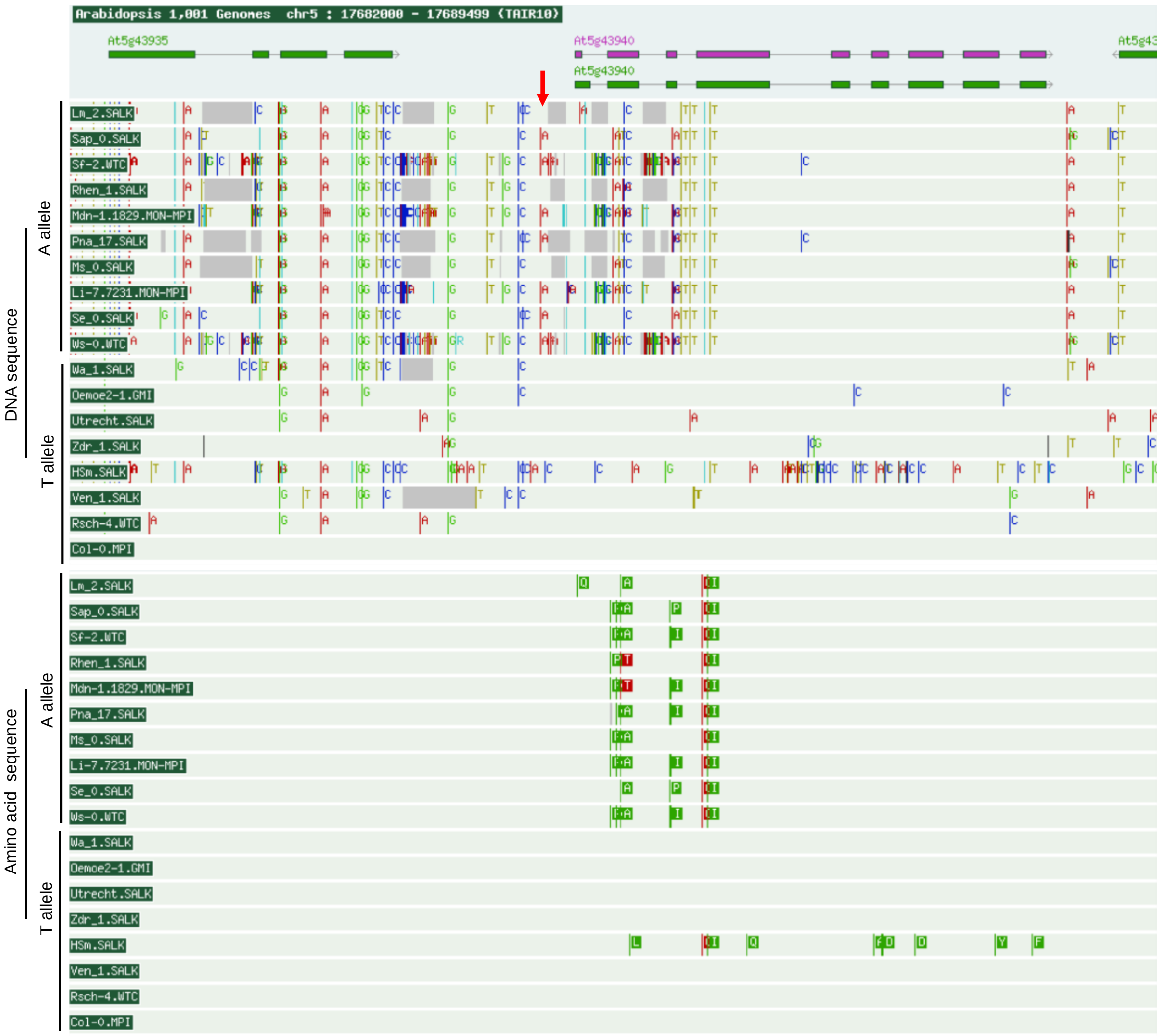

Supplementary Figure 4. Sequence analysis of natural *GSNOR* variants. Comparison of DNA and amino acid sequences of *GSNOR* (At5g43940) in phenotypically contrasting accessions grouped by their lead SNP variant. Genome sequences from chromosome 5: 17682000 bp to 17689499 bp covering the *GSNOR* gene model were shown in 1001 *Arabidopsis* genome browser (http://signal.salk.edu/atg1001/3.0/gebrowser.php)[61]. SNPs or codon changes relative to the Col-0 reference sequence are indicated by letters. Red arrow indicates position of lead SNP.

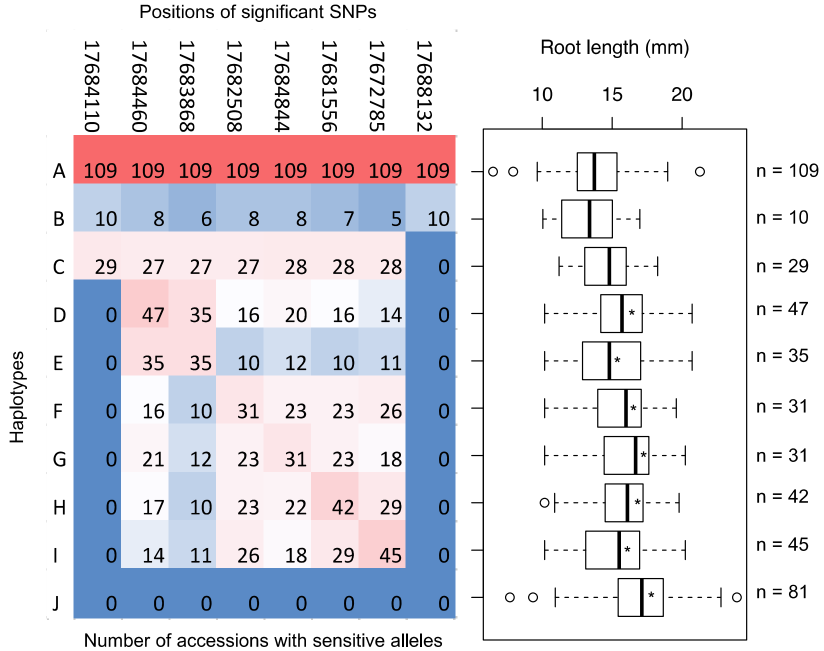

Supplementary Figure 5. Haplotype analysis of natural *GSNOR* variants. Haplotype analysis (left panel) of the significant eight SNPs identified by GWAS at high Fe in Figure 1B and box plots (right panel) for root length in accessions that are grouped by different haplotypes. The heatmap shows the number of accessions with sensitive alleles (that were defined by shorter roots in Supplementary Figure 3c) at different significant SNPs. Asterisk (*) indicates the significant difference between haplotype A and other haplotypes by Student's *t*-test ($p < 0.05$). The number (n) of accessions for each haplotype are shown in the figure. The source data are provided in a Source Data file.

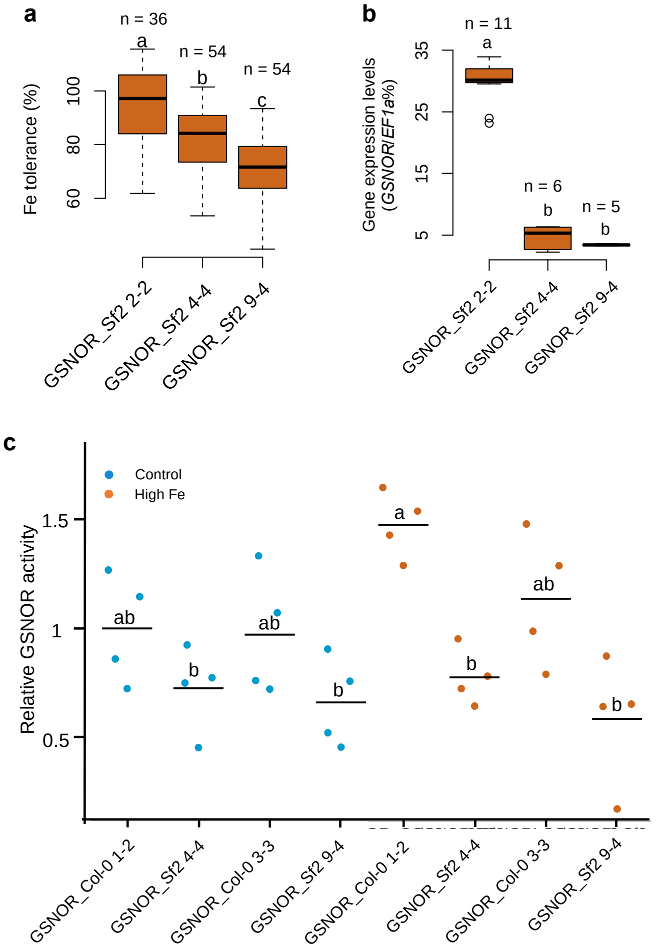

Supplementary Figure 6. Fe tolerance, transcript levels and enzyme activity of different GSNOR variants. Box plots for root tolerance to high Fe (**a**) and *GSNOR* transcript levels (**b**) in T3 generation of different T-DNA lines of *GSNOR_Sf*-2 construct. Fe tolerance (%) is defined by normalizing the root length at high Fe (½ MS with 350 µM Fe) with the root length at control (½ MS). n denotes the number of seedlings in (**a**) and the number of biological samples in (**b**). **c**, GSNOR activity in T3 generation of different T-DNA lines of *GSNOR_Col-0* and *GSNOR_Sf*-2 constructs. The seedlings were grown on the control and high Fe for 8 days. The value is presented as the mean and dot plots (n = 4 biologically independent samples). Two-way ANOVA analysis suggested a significant difference of GSNOR activity between the different genotypes ($p < 0.001$), but not for high Fe treatment and its interaction with the genotypes. Different letters indicated the significant difference at *p*-value <0.05 level by a two-way ANOVA analysis with Tukey's HSD test. The source data of Supplementary Figure 6a-c are provided in a Source Data file.

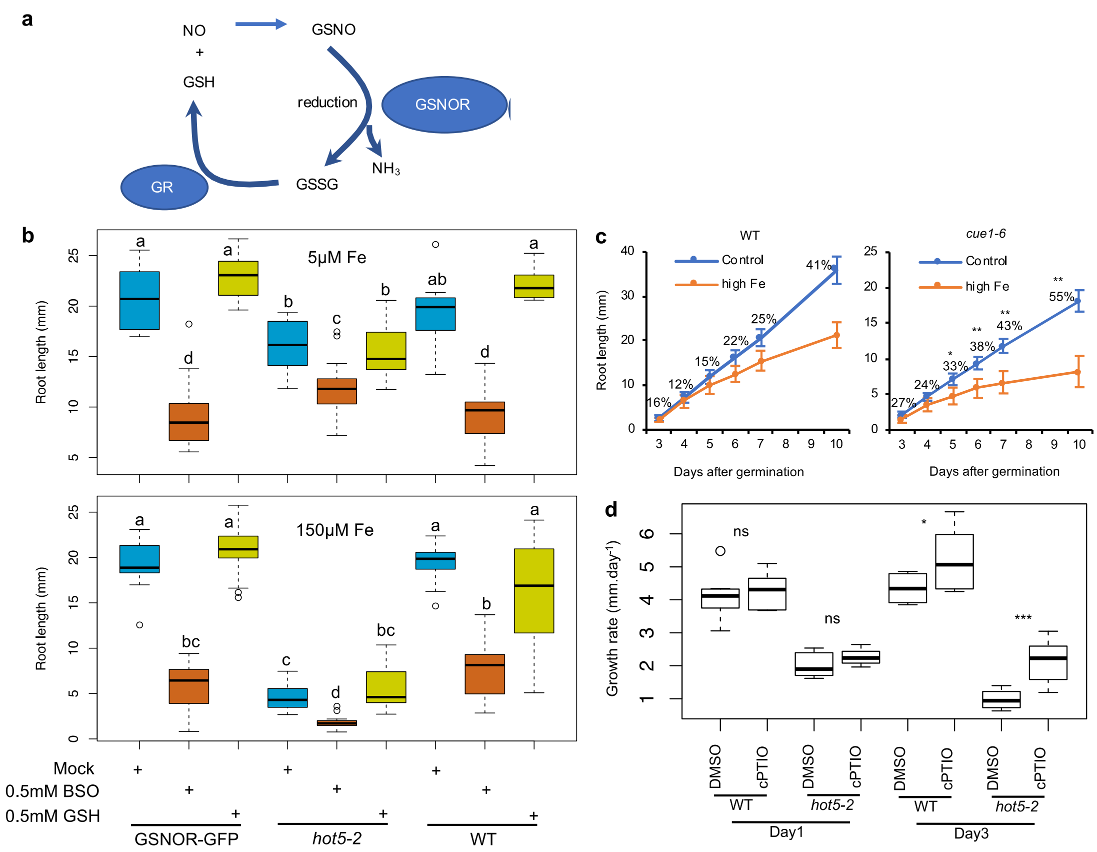

Supplementary Figure 7. High accumulation of nitric oxide causes root growth sensitivity to high Fe in *gsnor* mutants. a, Diagram of *GSNOR*'s involvement in nitric oxide (NO) metabolism. **b**, Box plots for root length of WT, *hot5-2* and *pGSNOR:GSNOR-GFP* complemented *hot5-2* lines upon glutathione (GSH), L-Buthionine-sulfoximine (BSO, an inhibitor of GSH synthesis), and Fe treatments 7 days after plating. Different letters indicate a significant difference according to two-way ANOVA with Tukey's HSD test at $p < 0.05$ level (n = 15 biologically independent samples). **c**, Mean root growth of NO overaccumulation mutant *cue1-6* and WT Col-0 in control and high Fe conditions. Error bars: standard deviation. n =18 biologically independent samples. Asterisk * and ** indicate significant differences of the inhibition on the root growth by high Fe between Col-0 and *cue1-6* on a given day at p <0.05 and p <0.01 respectively (Student's *t*-test). Percentage of root length at high Fe compared to the control for each genotype is indicated in the figure. **d**, Box plots for root growth rate of *hot5-2* mutant at high Fe and application of NO scavenger cPTIO. The 5-day-old seedlings grown in normal condition were transferred to 150 μM Fe with DMSO and 200 μM cPTIO for 1 or 3 days. Asterisk ***, * and ns indicated the significant difference at p < 0.001, 0.05 and no significance (ns) according to Student's *t*-test (n = 8 biologically independent samples). The Source data of Supplementary Figure 7b-d are provided in a Source Data file.

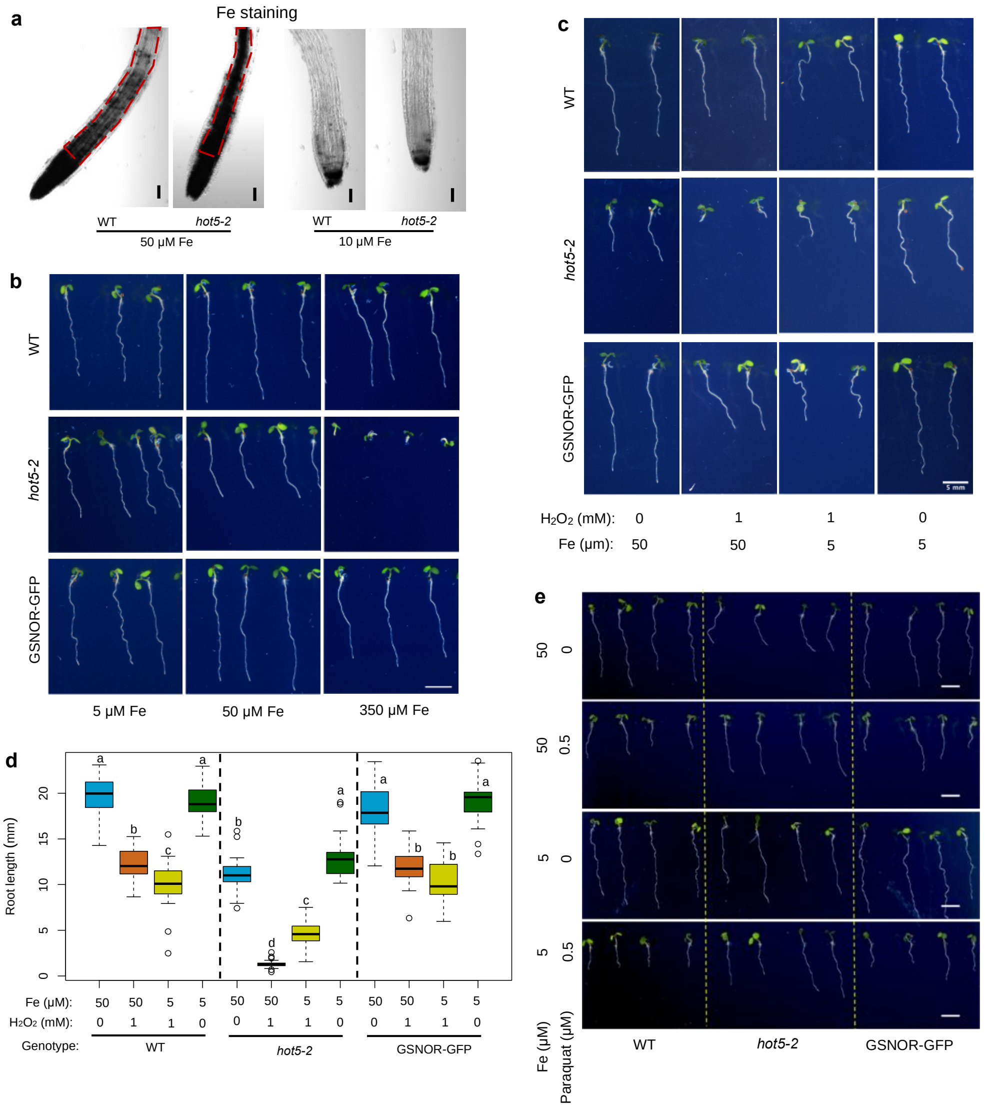

Supplementary Figure 8. *GSNOR* protects root growth from Fe-dependent oxidative stress but increases susceptibility to another oxidative stress inducer paraquat. a, Perls/DAB staining of WT and *hot5-2* root tips at day 5 after germination at low Fe and control conditions. The area surrounded by red dash lines indicates the area of differential Fe accumulation in the root tips of wild-type and *hot5-2*. **b**, Root growth of WT, *hot5-2* and *pGSNOR:GSNOR-GFP* complemented *hot5-2* lines at different Fe concentrations at day 6 after germination. Scale bar: 5 mm. **c**, Root growth of WT, *hot5-2* and *pGSNOR:GSNOR-GFP* complemented *hot5-2* lines upon H_2O_2 and Fe treatments 7 days after germination. **d**, Box plots for root length in experiments depicted in (**c**). n = 24 biologically independent samples. **e**, Representative seedlings of *gsnor* mutant line, the respective WT control and complemented lines at different concentrations of Fe and Paraquat at day 7 after germination. Scale bar: 50 µm in (a); 5 mm in (**b**, **c**, **e**). The source data of Supplementary Figure 8d are provided in a Source Data file.

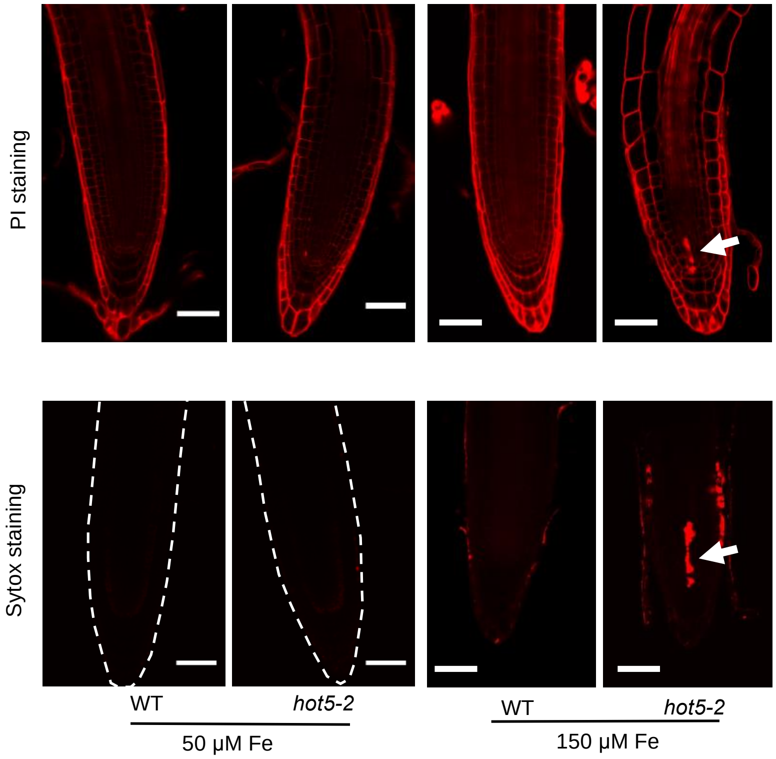

Supplementary Figure 9. *GSNOR* suppresses Fe-induced cell death in root meristems. Medial optical section of a representative root of *gsnor* mutant line and the respective WT control at 50 µM and 150 µM Fe at day 6 after germination using PI and Sytox Orange staining. The dotted lines outline the contour of the root tip. White arrow indicates areas of cell death. Scale bars: 50 µm.

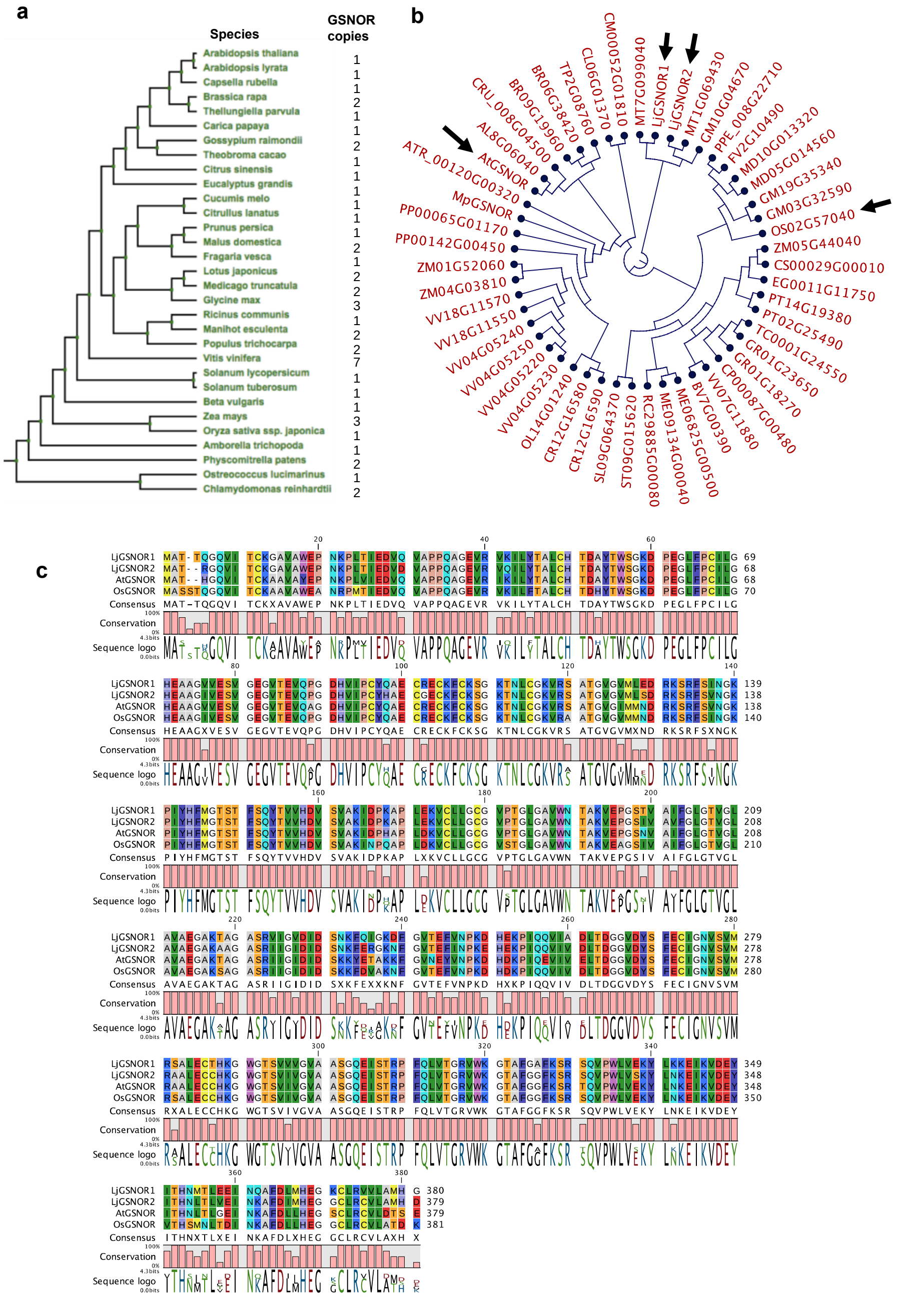

Supplementary Figure 10. Phylogenetic analysis of *GSNOR* among different plant species. a, Copy number of *GSNOR* in 31 species. These 50 genes include 49 genes in subfamily *ORTHO03D001554* in 31 species and a second copy of *GSNOR* found in *Lotus japonicus*. The results were analyzed in Plaza_V3 (https://bioinformatics.psb.ugent.be/plaza/versions/plaza_v3_dicots/genes/view/AT5G43940). **b**, Phylogenetic analysis of 51 *GSNOR* genes including 50 genes from (**a**) and one from *Marchantia polymorpha*. **c**, Alignment of GSNOR amino acid sequences from *Arabidopsis thaliana*, *Lotus japonicus* and *Oryza sativa*.

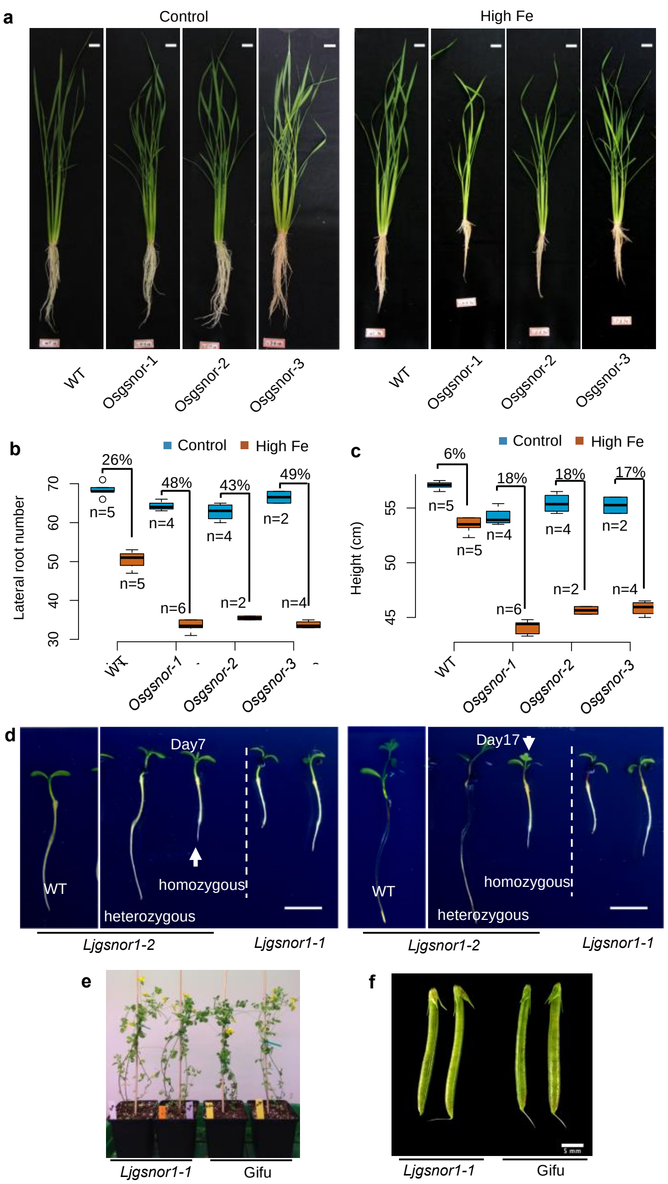

Supplementary Figure 11. Function of *GSNOR* in high Fe tolerance and development among different plant species. a, Representative whole seedlings of three independent *OsGSNOR* knockout lines as shown in Fig. 7b. **b**, **c**, Box plots for the number of lateral roots and shoot height in three independent *OsGSNOR* knockout lines shown in (**a**). The number (n) of homozygous plants and % trait differences are indicated in figure. **d**, Seedling phenotypes of representative *Ljgsnor1-1*, *Ljgsnor1-2* and WT plants 7 and 17 days after germination (transfer to 350µM Fe occurred at day 3 after germination). Homozygous seedlings are indicated with white arrows. **e**, Mature shoot phenotypes of representative *Ljgsnor1-1*, *Ljgsnor1-2* and WT plants grown in the standard soils in green house for about 3 months. **f**, Silique phenotypes of *Ljgsnor1-1* and Gifu grown in standard soils. Scale bars: 2 cm in (**a**), 5 mm in (**d** and **f**). The source data of Supplementary Figure 11b and 11c are provided in a Source Data file.

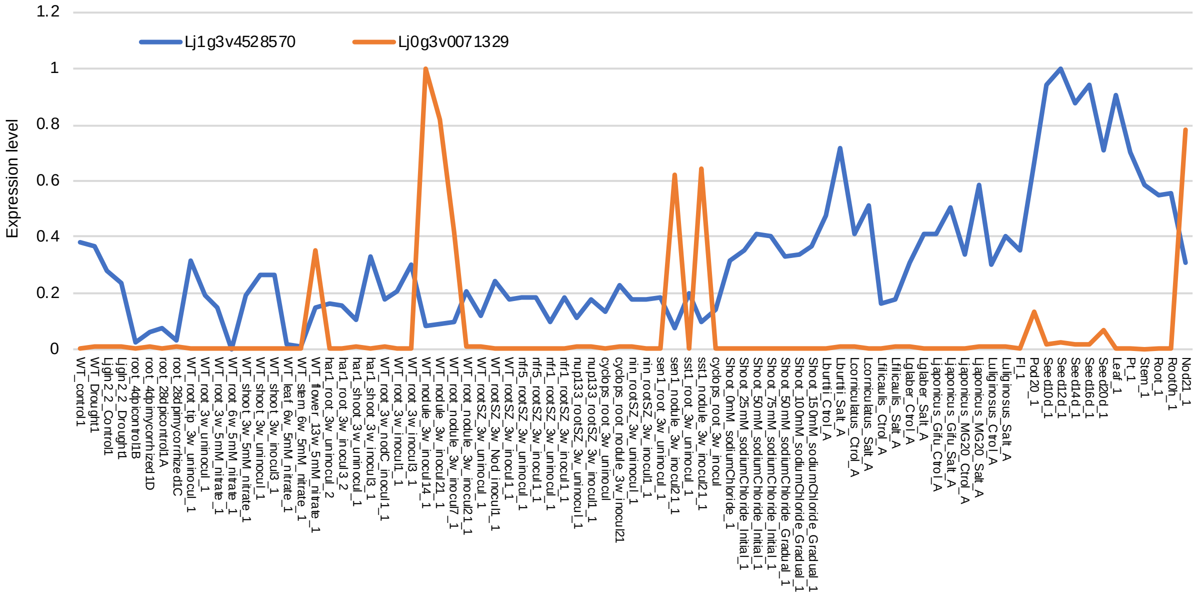

Supplementary Figure 12. Gene expression patterns of *LjGSNOR1* and *LjGSNOR2*. LjGSNOR1: Lj1g3v4528570; LjGSNOR2: Lj0g3v0071329.The data was download from the LjGEA project (entire LjGEA dataset by gene ID, normalized across condition / by row) (https://lotus.au.dk/expat/). The source data are provided in a Source Data file.

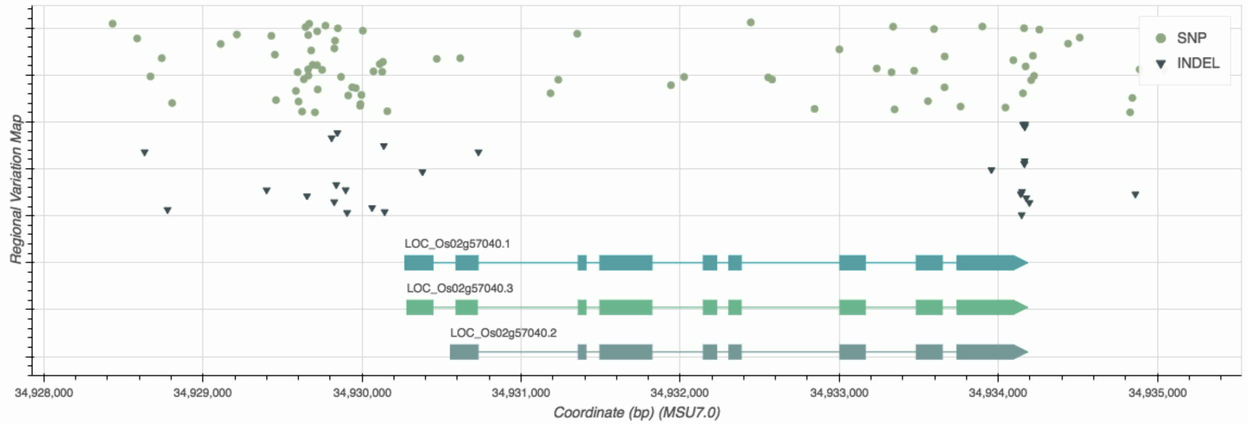

Supplementary Figure 13. Natural variants of *GSNOR* in rice. 112 variations including SNPs and INDELs were found between 2.0 kb upstream and 1.0 kb downstream of this gene (OS02G57040/LOC_Os02g57040) from 4729 accessions using RiceVarMap 2.0(http://ricevarmap.ncpgr.cn/v2/vars_in_gene/) using default parameters.

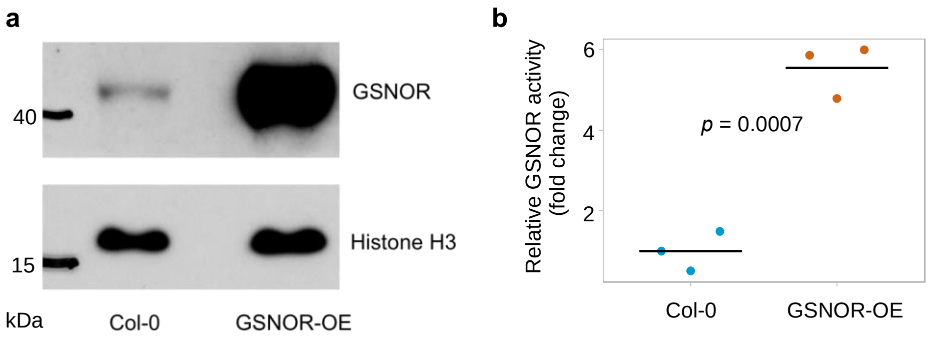

Supplementary Figure 14. GSNOR protein level and GSNOR activity in the *35S:GSNOR* line. a, Western blot analysis of GSNOR in the 7-day-old seedlings of Col-0 and *35S:GSNOR* (*GSNOR-OE*) line. A histone H3 protein is shown as a loading control. **b**, GSNOR activity assay of GSNOR in the 8-day-old seedlings of Col-0 and *GSNOR-OE*. The mean and dot plots of relative GSNOR activity was shown (n = 3 biologically independent samples). The seedlings were grown in ½ MS medium. *p*-value of student's *t*-test is shown. The source data are provided in a Source Data file.

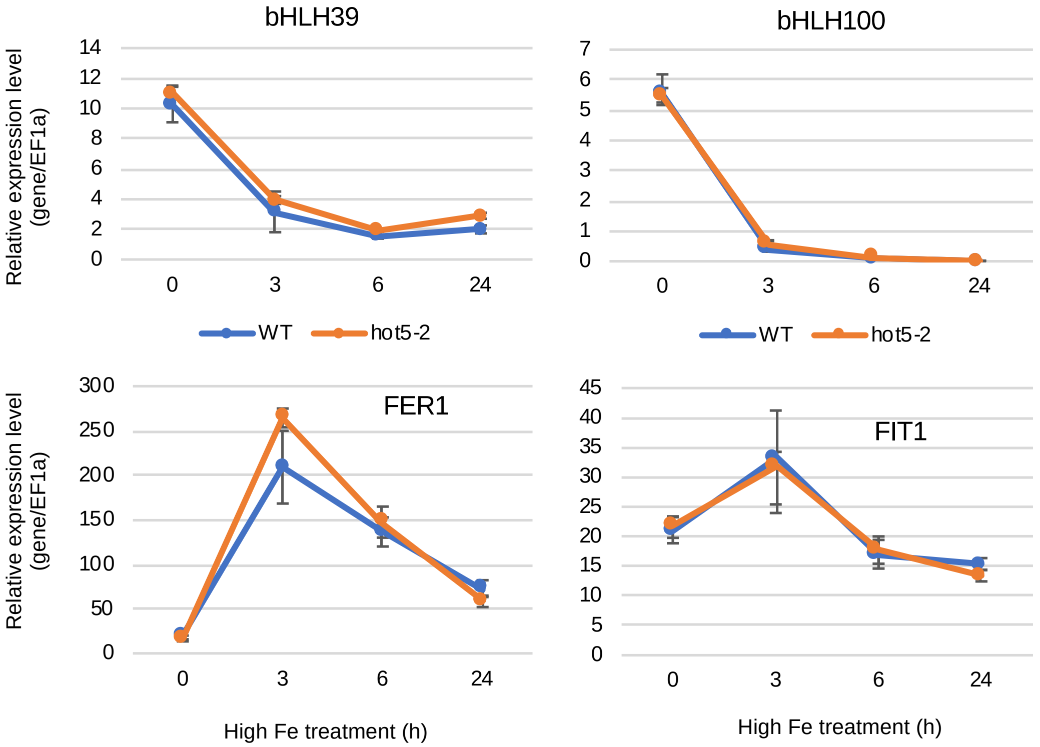

Supplementary Figure 15. Expression patterns of Fe deficiency responsive and Fe binding/storage marker genes in Col-0 and *gsnor* mutant *hot5-2*. Root samples were collected from seedlings grown in low Fe condition (1/2 MS containing 10 µM Fe) for 6 days, and then transferred to high Fe (1/2 MS containing 350 µM Fe) for 3 h, 6 h and 24 h. RNA was extracted from these samples for qPCR analysis. The expression value of each gene was normalized to the *EF1a* gene and shown as the mean ± standard deviation (n = 6). Three biologically independent samples and two technical replicates for each sample were measured for each gene. The source data are provided in a Source Data file.

Supplementary Table 1. Broad sense heritability estimated for variation of primary root length in 319 *Arabidopsis* accessions grown in the control and high Fe conditions.

Broad sense Heritability	Day3	Day4	Day5	Day6	Day7	Day8	Day10	Day13
Control	0.442	0.478	0.442	0.474	0.483	0.494	0.523	0.522
High Fe	0.411	0.428	0.443	0.46	0.463	0.492	0.492	0.478

The source data are provided as a Source Data file.

Supplementary Table 2. List of primer sequences used in this study.

Primer ID	Sequence 5'-3'	Purpose
GSNOR_Col_F	GCTTGATATCGAATTTGATTGATGCTAAACCTCAG	Cloning
GSNOR_Col_R	CGGGCTGCAGGAATTGTAAACTATATGATTAGACATG	Cloning
GSNOR_Sf2_F	GCTTGATATCGAATTTGATTGATGCCAAACCTCAG	Cloning
GSNOR_Sf2_R	CGGGCTGCAGGAATTGTAAACTATATGATTAGACATG	Cloning
30033535_F	CCCTGCAGCTTCATGGCCAAGGATA	Genotyping
30033535_R	TGTGTTGTGTTGTGGTGTCTCACTCTCA	Genotyping
30060068_F	TGCAACCACCCCAAAATGGAAAAGC	Genotyping
30060068_R	TGATCTCACCGATGGTGGGGTTGA	Genotyping
30075087_F	CAAGCCCAACAGTTCCAAGGCCAA	Genotyping
30075087_R	TGCGGTTGCGGGGAACACTTAAAA	Genotyping
OsGSNOR_sgRNA	GCTGAGGGGGCAAAATCAGC	CRISPR/Cas9
Osgsnor-F	ACATACCATTGCCTCGCACA	Genotyping/sequencing
Osgsnor-R	CCACCAGACATGGATTGCCT	Genotyping/sequencing
GSNOR_F	TGGCACTGTTGGACTTGCTGTTG	qPCR
GSNOR_R	TGGCTTGTCGTGATCCTTTGGG	qPCR
EF1a_F	CCTTGGTGTCAAGCAGATGA	qPCR
EF1a_R	TGAAGACACCTCCTTGATGATTT	qPCR
bHLH39_F	TGCCTCTGGCCAATCGAAGAAG	qPCR
bHLH39_R	TGTACTTCAAGCTTCGAGAAACCG	qPCR
BHLH100_F	CTTCCTCCCACCAATCAAACGAAG	qPCR
BHLH100_R	ACTTGCTCTTGCAGCTCTGGTATG	qPCR
FIT1_F	AGCTCTCCTTCTCCGGACACATAC	qPCR
FIT1_R	GCTCTGTTCGAAGCATGTCCCATC	qPCR
FER1_F	CAACGTTGCTATGAAGGGACTAGC	qPCR
FER1_R	ACTCTTCCTCCTCTTTGGTTCTGG	qPCR
GSNOR_SF-2_LP 2	GGTTCCCAGTCTAGCTACGT	Sequencing
GSNOR_SF-2_LP 3	CCTCGCACTCTCACTATCTGT	Sequencing
GSNOR_SF-2_LP 4	ACAGGAGTTCAAGCTGGAGA	Sequencing
GSNOR_SF-2_LP 5	GTCCTTCTCTTCTTTCTTTGCGA	Sequencing
GSNOR_Col_RP1	GTCGGGTCGGGTCGTTAATA	Sequencing
GSNOR_Col_RP2	AGGACACAGCCTCAAATTGA	Sequencing
GSNOR_Col_RP3	GGTGGCAATTCTTACCAGTGG	Sequencing
GSNOR_Col_RP4	TGCCAATGATCCTTGAAGCAC	Sequencing
GSNOR_Col_RP5	GCACTTGGGTTCGACTCTTG	Sequencing
