# Supplementary material for: GSNOR provides plant tolerance to iron toxicity via preventing iron-dependent nitrosative and oxidative cytotoxicity
Source: Nat Commun. 2019 Aug 29;10:3896. doi: 10.1038/s41467-019-11892-5 (PMC6715714; doi:10.1038/s41467-019-11892-5)
Supplement: Supplementary file 2 — Peer Review [file 41467_2019_11892_MOESM2_ESM.pdf]

Reviewers' comments:

Reviewer #1 (Remarks to the Author):

In this manuscript entitled 'GSNOR confers plant tolerance to iron toxicity via preventing iron-dependent nitrosative and oxidative cytotoxicity', Busch and colleagues report the identification of a major QTL for tolerance to Fe-toxicity in Arabidopsis and find that this QTL encodes GSNOR1, a key enzymes degrading S-nitrosoglutathione (GSNO). Subsequent studies reveal that variable transcription levels in different accessions are the main cause for tolerance to Fe-toxicity. The authors further suggest that GSNOR1 is involved in the inhibition of Fe-dependent nitrosative and oxidative stresses. Similar regulatory mechanisms are also functional in rice and Lotus japonicus.
The reported discoveries are interesting, revealing an additional layer of regulatory roles of the highly conserved GSNOR genes. Overall, the genetic studies are well designed and executed. However, the molecular or biochemical basis of the GSNOR-mediated tolerance to Fe-toxicity remains elusive and the study is therefore short of mechanistic understanding of the reported physiological response.
NO has been shown to antagonize the toxic effects of ROS via various mechanisms. Therefore, it is unlikely that ROS is a factor, at least a major factor, of the GSNOR-dependent tolerance to Fe-toxicity. The authors' claim 'the protective role of GSNOR may be specific to Fe-dependent H2O2-mediated oxidative toxicity' is a pure speculation. Instead, it has long been knows that Fe and reactive NO can directly react to produce vraious compounds, some of which are toxic to cells (for example, Rahmanto et al., JBC, 287: 6960 and references therein). Alternatively, excessive NO in gsnor1 mutants causes hyper-nitrosylation of key players of Fe homeostasis, resulting in cellular toxicity.

Other comments
--Several experiments lack appropriate controls.
--The writing of the paper could be further improved.
--Several key references are incorrectly cited.

Reviewer #2 (Remarks to the Author):

The authors characterize the GSNOR gene related to NO metabolism that was identified from GWAS for Fe tolerance evaluated by root growth in Arabidopsis. Overall, the methods and results were significant and interesting. I have several significant concerns.
1) GWAS session;
The authors mainly showed phenotypes of absolute root length under high Fe concentration with root length data under control condition for the evaluation of Fe tolerance thorough the manuscript. But relative root length (Fe/Control) were also used as a Fe tolerance at their GWAS. GWAS for root length with high Fe detected the strongest association. On the other hand, we can see the large natural variation of root length under control condition. Hence, relative root length, which is defined as a Fe tolerance by the authors, should be presented in the main story. In that case, was the lead SNP changed? Why do you focus on the 10 day's GWAS? Day 13 has stronger association than other time points.
Multi-trait GWAS was conducted in this manuscript. But it is not likely required to explain their single prominent peaks. Could you kindly explain the efficacy and effect of the results?
They performed GWAS using GWAPP/GWA-portal. I believe that it might be done by default setting. I think that it is not sufficient explanation for the readers.
Hb2 was shown in only loot length under high Fe, Hb2 in control and Fe tolerance can be calculated. And we cannot say that the value under 0.5 is high heritability.
What is the percentage of the proportion 20% from the fig S2c? It seems to be small to explain the strong association.
Supporting Figure S1a should be zoomed in for the image to see the accessions.

2)Allelic variation session;
GWAS significant peaks were detected in the only high Fe and Fe tolerance, not in control. But the authors used the public expression data among some accessions, which is different from GWAS sample and condition, to select candidate causal gene. The variation of expression under the Fe condition in roots would be better to identify the candidates. If GSNOR was explained by expression level polymorphism, the promoter variant such as fig S4 is supposed to be associated with the expression level of accessions. Are the expression level and difference of expression inducible by Fe/constitutive?
The tolerance phenotype of overexpression GSNOR in fig 3b is hard to observe as described in line 17-18 page5.

3)NO and H2O2 session;
The authors tried to analyze GSNOR function under the high Fe using the knockout mutant. They showed the NO metabolism and inducible cell death determined by GSNOR function. However, they are poorly distinguished. NO, GSNO and GSH production can be measured. Additionally, evaluation of cell death and Fe accumulation should provide convincing evidence. Is there any difference of the phenotype related to GSNOR activity among the accessions? Were GSNOR expressed in the root meristem, in which NO accumulation and cell death occurred as the authors mentioned?
Some publications have reported that Fe deficiency and Cd induce NO accumulation including GSNO/GSNOR. How was the high-Fe dependent NO oxidative stress explained?
Supporting Fig 6c should be test the significance between the WT and the mutant.
Page 8 line 8; how is the difference with "Fe enhance H2O2 damage"
Page 8 line11; Why were the KO tolerant to paraquat? Are the other toxic metals inducing ROS inhibit the growth of the KO?

4) higher plants session;
The authors mentioned that the aim and result of this study is to help breeding in discussion and background. And also Arabidopsis transgenic line introduced high expression type GSNOR (Col-0 accession) showed the high shoot FW and slightly, but significant, long root length compared to low expression type. Furthermore, the authors used word of "GSNOR confers tolerance". Overexpression line or high expression line/accession should be tested rather than knockout mutants.
Supporting fig10e and f were hard to follow.

5) Discussion
Page11 line 14: GSNOR of human and other species would be compared in supporting fig 9.
I think the discussion would argument from the study of NO metabolism related to GSNOR (GSNO) involved in Fe deficiency and expression/promoter polymorphism explaining variations on the consequence of what authors find.

Reviewer #3 (Remarks to the Author):

This paper tackles the issue of high iron (Fe) toxicity in plants through screening for genes that confer tolerance to high Fe in Arabidopsis thaliana accessions. Using a GWAS approach, the authors clearly demonstrate that significant variation in Fe tolerance is associated with variant alleles of S-nitrosoglutathione-reductase (GSNOR). This extensive study involves competent, high resolution phenotyping and GWAS statistical analysis, supported by allelic complementation, leaving no doubt that the relevant genetic locus conferring a significant percentage of Fe tolerance has been identified. They go on to provide evidence that GSNOR also confers tolerance to high Fe in rice and Medicago

truncatula, expanding the significance to crop plants. The authors state that previous attempts to identify genes involved in tolerance to Fe have not been successful, making their study a landmark in this field and identifying a gene for potential targeted breeding efforts. The actual mechanism by which GSNOR confers Fe tolerance is not illuminated here, although it is linked to the over-accumulation of NO species through studies of Arabidopsis plants that carry a null mutation in GSNOR. Further investigation of the mechanism is clearly outside the scope of the present study.

The authors could better strengthen the significance of the work to agriculture if it were possible to relate the Fe levels used for their studies to those causing Fe toxicity in the field. Statements about Fe toxicity in the field are very general, and overall not particularly informative. In addition, as shown in Fig. 3b, attempts to increase GSNOR expression conferred only minor change in Fe toxicity. The authors do comment that could be due to the overall high resistance to Fe of the Arabidopsis accession tested. However, it would be more satisfying it the authors actually showed levels of the GSNOR protein in this and some of their other experiments, which is readily accomplished with available GSNOR antisera. In addition, GSNOR activity can be measured in whole cell extracts.

Overall, the authors have presented an excellent study that is a roadmap for using GWAS to discover novel genes associated with specific phenotypes.
Additional issues that should be addressed are listed below:
1) The authors may wish to indicate at first introduction, the accession background of the hot5-2 and hot5-4 mutants, as this is relevant to their subsequent introduction of the A and J haplotype alleles into hot2-4.
2) Fig. 3b. The authors should either correct the scale of the hot5-2 graph to correspond to the scale of the other graphs, or express all values as a percentage of the growth of the control (50 uM fe?).
3) As mentioned above - Fig. 5c. It would be useful to have tested the level of GSNOR protein in these lines. This may provide insight as to whether OE of GSNOR is indeed occurring. Activity of GSNOR can also be tested in seedlings. This is particularly relevant to their discussion, Page 10, lines 276 to 282 and to the possibility of engineering tolerance using this enzyme.
4) Please state more specifically in the text what the inhibitory mechanism of BSO.
5) Fig. 6e. The bottom of the X-axis legend is partially covered by the panel g.
6) Supp. Fig. 7a. It is not clear why the authors have a red arrowhead in the wt picture at 50 uM Fe, but not in the mutant picture. I believe the goal here is to show more accumulation in the mutant, but this single picture makes that difficult to confirm.
7) Supp. Fig. 7d. The indication of H2O2 levels has extra "0"s that need to be removed.
8) Supp. Fig. 10. The Ljgsnor1-1 picture presented for Day 7 and day 17 appear identical. I believe the authors have made an error in constructing this figure.
9) Page 9. Line 256 "that present in Arabidopsis gsnor mutants" should be "that are present in Arabidopsis gsnor mutants". This result would also be better explained if the authors added more specifics as to the phenotypes to which they are referring - that is increased branching and reduced fertility.
10) Concerning subfunctionalization of the the putative two GSNOR genes in Lotus, it should be possible for the authors comment on whether or not these two genes are similarly expressed. They might also consider that stating more specifically that it could be differences in substrate specificity of GSNOR for substrates other than GSNO. Can the authors also specifically state that both genes are in the ADHIII clade, not the ADHI clade?

Grammatical or other issues:
1) Page 3. Line 55 – "sensitive of primary" should be "sensitivity of the primary"
2) Page 5. Line 108 "and no root" should be "and showed no root".
3) Page 5. Line 110 "while only 20% inhibition in" should be "while only 20% inhibition was observed in".
4) Page 9. Line 238 "retarded much more pronounced" should be "retarded much more"
5) Page 9. Line 225 "of Ljgsnor1 mutants at the visible development defects" – seems it should be "in Ljgsnor1 mutants of visible development defects"

6) Page 11. Line 304. “It is widely accepted that Fe toxicity highly dues to generate hydroxyl radical via the Fenton reaction with H2O2”. This sentence makes no sense.
7) Page 11. Line 308. “prevents from cell death” should be “prevents cell death”
8) Page 11. Line 309. “to generating” should be “for generating” or “to generate”
9) Page 11. Line 312. RNS is introduced for the first time with no definition.
10) Page 11. Line 315. “reduction that” should be “reduction, which” in order to make this sentence easier to follow.

Reviewer #4 (Remarks to the Author):

Genes are known to be differentially expressed by high iron stress. However, a genetic trait explaining tolerance to iron toxicity is not known, even though iron toxicity represents an agricultural problem for rice on waterlogged acidic soils in Africa and Asia. This manuscript proposes that GSNOR gene expression variation can explain tolerance to high iron apparent as decreased root growth inhibition, and authors suggest that decreased NO in the presence of elevated GSNOR and Fe is the cause for reduced oxidative stress in the root meristem.
In a first part of the manuscript the authors describe GWAS studies using in total several hundred natural variation lines and transgenic Arabidopsis plants to identify and confirm the high iron root length and gene expression phenotype. In a second part the authors investigated the cause of the phenotype using physiological experiments with different transgenic lines, grown in the presence or absence of iron and NO to study the connection of high iron, GSNOR expression and root length. Finally, the authors show in transgenic rice and Lotus loss-of-function plants that low GSNOR expression under high iron is also associated with reduced root growth in other species.

Overall, the findings are interesting and novel in the context of root development and iron and fit to the currents efforts to identify genetic variation traits to high iron or more particularly to root growth inhibition. However, regarding the discussion and importance of the results in terms of agricultural high iron tolerance in Arabidopsis and in crops, major questions remain and here more clear data and analyses are needed to back up the claims that authors want to make in this context.

Major comments:

1) To claim that GSNOR gene expression contributes to high iron toxicity tolerance, authors should investigate plant development in general, leaf bronzing, a typical symptom of iron toxicity, flowering phenotypes, seed production, in Arabidopsis and also in rice. It remains unclear what is the effect of the root growth trait on general growth and yield of the plants (this also accounts for the introduction, which does not comprise major adaptive effects known to confer high iron tolerance). All analyzed phenotypes were restricted to root growth, especially in the early plant developmental stages. Plants might have adapted in other ways to high iron and the GSNOR effect might only be relevant transiently for some root growth adaptation but may not contribute to overall plant tolerance.
2) In the same line, there are some studies conducted with high iron transcriptome changes and tolerance in rice. The authors should check and discuss whether GSNOR gene expression variation has been found in any of these studies.
3) Physiological experiments are rather narrow and merely address iron toxicity at the root apex leading to different root lengths of primary roots. Other aspects of NO and iron effects are not experimentally addressed and not even discussed. For example, it has been shown in several studies that NO has a positive effect on ethylene, that both NO and ethylene promote iron uptake via regulation of transcription factors, which is a response to NO along the root and in the root hair zone. Furthermore, NO can affect regulation of ethylene synthesis and perhaps other components relevant for iron uptake regulation via nitrosylation. No studies are presented to investigate the effect of GSNOR expression on Fe acquisition regulation in roots at the molecular level.
4) In this context, it would be interesting to know how iron uptake itself is actually affected and what

are the iron contents of plants in different organs. Principally, one might assume that short root growth is an advantage in the presence of high metals rather than longer roots, which would have a higher surface for toxic metal uptake. Thus, it seems important for iron tolerance to consider mechanisms that restrict iron uptake into the root cells and restrict long-distance transport of iron to shoots and seeds. So what is the actual effect of GSNOR expression here?
5) The authors make the point that high Fe causes nitrosative cytotoxic stress. This raises the question whether it is not possible to prove such effects and detect the effective compounds in plant cells.

Additional comments:

1) How was it controlled by authors that Fe is soluble at 350 μM under the respective pH? Is Fe taken up and can it be measured that plants differ in Fe contents under normal and high iron?
2) The authors should describe in earlier paragraphs what was exactly the variation, which phenotypes were observed.
3) Explain better T-and A-alleles in the text.
4) Explain earlier in the text how GSNOR expression correlates with the phenotype.
5) Explain better the use of different statistical methods for the physiological assays and correct it in the figure legends.

We thank the four reviewers very much for the time, effort, thought and constructive comments on our manuscript. We have considered the comments, concerns and suggestions and revised the manuscript to address these. Please find our point-by-point responses below. The paragraphs in regular font are the comments from reviewers, and the paragraphs in italic are our responses. We also highlighted edits in the revised manuscript with track changes. We believe our revisions significantly improved the quality of our manuscript and hope that the reviewers agree.

Reviewers' comments:

Reviewer #1 (Remarks to the Author):

In this manuscript entitled 'GSNOR confers plant tolerance to iron toxicity via preventing iron-dependent nitrosative and oxidative cytotoxicity', Busch and colleagues report the identification of a major QTL for tolerance to Fe-toxicity in Arabidopsis and find that this QTL encodes GSNOR1, a key enzymes degrading S-nitrosoglutathione (GSNO). Subsequent studies reveal that variable transcription levels in different accessions are the main cause for tolerance to Fe-toxicity. The authors further suggest that GSNOR1 is involved in the inhibition of Fe-dependent nitrosative and oxidative stresses. Similar regulatory mechanisms are also functional in rice and Lotus japonicus.

The reported discoveries are interesting, revealing an additional layer of regulatory roles of the highly conserved GSNOR genes. Overall, the genetic studies are well designed and executed. However, the molecular or biochemical basis of the GSNOR-mediated tolerance to Fe-toxicity remains elusive and the study is therefore short of mechanistic understanding of the reported physiological response.

Question: NO has been shown to antagonize the toxic effects of ROS via various mechanisms. Therefore, it is unlikely that ROS is a factor, at least a major factor, of the GSNOR-dependent tolerance to Fe-toxicity. The authors' claim 'the protective role of GSNOR may be specific to Fe-dependent H2O2-mediated oxidative toxicity' is a pure speculation. Instead, it has long been known that Fe and reactive NO can directly react to produce vraious compounds, some of which are toxic to cells (for example, Rahmanto et al., JBC, 287: 6960 and references therein). Alternatively, excessive NO in gsnor1 mutants causes hyper-nitrosylation of key players of Fe homeostasis, resulting in cellular toxicity.

Reply: *As outlined by the reviewer, NO has been shown to antagonize the toxic effects of ROS in many studies. In addition, NO can also interact with H_2O_2 thereby causing a stronger cytotoxic effect through an unknown mechanism as observed in previous studies in plant disease response and cell death in leaves*

(Delledonne et al., 2001; Lin et al., 2012) and also in Escherichia coli (Pacelli et al., 1995). While the gsnor mutant is tolerant to paraquat (Chen et al., 2009), which is a common oxidative stress inducer and converts oxygen (O_2) to the superoxide (O_2^-) radical (Dinis-Oliveira et al., 2008), it is very sensitive to both high Fe and Fe-dependent H_2O_2 cytotoxicity, in which Fe could reacts with H_2O_2 to generate $OH^\cdot$ (a highly active ROS) (Toyokuni, 1996; Galaris and Pantopoulos, 2008). Thus, we think it is reasonable to suggest that "the protective role of GSNOR may be specific to Fe-dependent H_2O_2-mediated oxidative toxicity, as the gsnor shoots and roots were tolerant to paraquat[34] (Supplementary Fig. 8e), another common oxidative stress inducer, which converts oxygen (O_2) to the superoxide (O_2^-) radical[38]", although we can't exclude other possibilities as suggested by the reviewer. To make this clear, we added the discussion "as the interplay of high Fe, ROS and reactive nitrogen species (RNS) is highly complex [46,47], it will be very interesting to investigate these interactions and their impact of cytotoxicity" in the revised discussion part[page 11, line 34ff.].

Other comments

Question: Several experiments lack appropriate controls.

Reply*: We thank the reviewer for pointing this out. Upon checking, we only discovered that we had omitted control data from Supplementary Figure 8 (now Figure S9), even though the control data was contained in Fig.6. We have corrected this omission.*

Question: The writing of the paper could be further improved.

Reply*: We aimed to improve the writing during our revisions and we are certain that the further editorial process at Nat. comm. will address potential writing related issues.*

Question: Several key references are incorrectly cited.

Reply*: Thanks for the comment. To cite the references more precisely, we added 10 new references in the revised manuscript, and corrected the order of references No.25 and No.26, and the omitted reference for No.61.*

Reviewer #2 (Remarks to the Author):

The authors characterize the GSNOR gene related to NO metabolism that was identified from GWAS for Fe tolerance evaluated by root growth in Arabidopsis. Overall, the methods and results were significant and interesting.

We thank the reviewer for their assessment and for the thorough review of our manuscript.

I have several significant concerns.

1) GWAS session;

Question: The authors mainly showed phenotypes of absolute root length under high Fe concentration with root length data under control condition for the evaluation of Fe tolerance thorough the manuscript. But relative root length (Fe/Control) were also used as a Fe tolerance at their GWAS. GWAS for root length with high Fe detected the strongest association. On the other hand, we can see the large natural variation of root length under control condition. Hence, relative root length, which is defined as a Fe tolerance by the authors, should be presented in the main story. In that case, was the lead SNP changed? Why do you focus on the 10 day's GWAS? Day 13 has stronger association than other time points.

Reply: *We thank the reviewer for pointing out these issues, which we have clarified the text now. For the GWAS analysis, using Day10 or Day13 did not affect the selection of candidate genes. We chose to focus on day10, as both the control and high Fe have the highest broad heritability compared to other days in this study (Supplementary Fig. 1c). Therefore, we used the data from Day10 as the representative point in GWAS analysis. To clarify this, we added to the text: "Moreover, we chose to focus on the day10 GWAS as the representative GWAS, as the broad heritability was highest at this day in control and high Fe conditions (Supplementary Fig. 1c)" [page 4, line 21ff.].*

Regarding the first concern of the reviewer, as shown in Figure 1a and b, the SNP peak was not changed by the absolute root length or relative root length at high Fe. The lead SNP of GWAS was changed from SNP17684100 to SNP17684460 (a synonymous-coding in GSNOR) when relative root length was used, but all significant SNPs are still in the same LD region that is tagged by the lead SNP, thus it did not affect the follow-up analysis. To clarify, we added in the text: "As the SNP peak was the same regardless of using the absolute root length or relative root length at high Fe, or the multi-trait GWAS, we decided to use the absolute root length at high Fe for further analysis" [page 4, line 19ff.].

Question: Multi-trait GWAS was conducted in this manuscript. But it is not likely required to explain their single prominent peaks. Could you kindly explain the efficacy and effect of the results?

Reply*: It is true that the multi-trait GWAS is not required the single prominent peaks. It is merely additional support for the association at the GSNOR locus that we had found using root length in high Fe or relative root length (high Fe/control). It therefore was providing additional evidence for the association of the GSNOR locus and high iron dependent growth variation using an independent GWAS*

approach that is able to partition the observed root growth variance as whether it was high-Fe dependent or shared between growth conditions.

Question: They performed GWAS using GWAPP/GWA-portal. I believe that it might be done by default setting. I think that it is not sufficient explanation for the readers.

Reply*: We thank the reviewer for this suggestion. We have added the sentence "GWAS was performed in GWAPP/GWA-portal by using default setting (no transformation and minor allele count (MAC) > 15)" in the Method in the revised manuscript [page 19, line 20ff.].*

Question: Hb2 was shown in only loot length under high Fe, Hb2 in control and Fe tolerance can be calculated. And we cannot say that the value under 0.5 is high heritability.

Reply: *Thanks for the suggestion. In the revision, Hb2 in control was added "The broad sense heritability of the observed variation ranged from 0.442 to 0.523 in control conditions and from 0.411 to 0.492 in high Fe conditions, with the highest value observed for day 10 (Supplementary Fig. 1c)" [page 4, line 11ff.]. Since data of individual seedling for each accession under both the control and high Fe was required for Hb2 calculation, this was not possible for a ratio and we thus didn't calculate Hb2 for Fe tolerance. We also removed the statement that a value under 0.5 is high heritability.*

Question: What is the percentage of the proportion 20% from the fig S2c? It seems to be small to explain the strong association.

Reply: *The percentage plotted on the y-axis of Fig S2c is the ratio of the root length of accessions grown at high Fe compared to that in the control.*

The p-value of GWAS indicates the probability for an association between the trait variation and SNPs. This EMMAX-based p-value does not scale with the effect size but it strongly indicates that the association is much larger than expected by chance, even when accounting for population structure.

To estimate the effect of the top SNP to explain the observed phenotypic variation, we used a multi-step GWAS. According to this, this genetic locus explained a notable proportion (20%) of the root growth variation at high Fe. Based on the literature, smaller proportions can be detected using GWAS in Arabidopsis panels – for instance recently Jia et al. 2018 a major peak explained 11.7% of the variation.

To clarify the fact that the 20% didn't refer to Fig. S2C(now Supplementary Fig. 3c), we now have reworded: "When conducting a conditional GWAS using the lead SNP:17684110, we found that only a single SNP still exceeded the Bonferroni-corrected threshold (Supplementary Fig. 3b). This genetic locus explained a notable proportion (20%) of the root growth variation at high Fe. The T-variant (54%) of this

lead SNP was associated with higher Fe tolerance and the A-variant (46%) was associated with lower Fe tolerance within these 319 accessions (Supplementary Fig. 3c)" [page 4, line 24ff.].

Question: Supporting Figure S1a should be zoomed in for the image to see the accessions.

Reply: *Thanks for this excellent suggestion. To zoom in Supplementary Fig. 1a, we have divided Figure S1 into two Supplementary figures in the revised manuscript.*

2)Allelic variation session;

Question: GWAS significant peaks were detected in the only high Fe and Fe tolerance, not in control. But the authors used the public expression data among some accessions, which is different from GWAS sample and condition, to select candidate causal gene. The variation of expression under the Fe condition in roots would be better to identify the candidates. If GSNOR was explained by expression level polymorphism, the promoter variant such as fig S4 is supposed to be associated with the expression level of accessions. Are the expression level and difference of expression inducible by Fe/constitutive?

Reply*: We agree with the reviewer that the expression of genes surrounding this SNP peak of different accessions under high Fe condition might be better than that from the public data under the control conditions, in particular if the causal gene would be induced by high iron treatment. However, even treatment data might not suffice as depending on the dynamics of the expression (genes can be expressed transiently) or on the cell-type expression pattern (genes might be expressed in just few cells and their expression in bulk samples might be below the sensitivity), it might be difficult to know or to catch the right timepoint or tissue quantity for the expression analysis. Fortunately, in our case the public expression data of accessions under the control condition was sufficient and supported by other data to indicate that GSNOR is better than others surrounding this SNP peak to be a candidate gene. Our follow-up experiments revealed GSNOR is indeed the causal gene. We nevertheless tried to increase clarity about the condition for which the expression data had been acquired in the text "We reasoned that a causal gene might be differentially expressed already in control conditions in Arabidopsis accessions containing different lead SNP variants" [page 5, line 2ff.].*

Consistent with this result, the expression of GSNOR from both high Fe resistant accession (Col-0) and high Fe sensitive accession (Sf-2) was not affected by high Fe treatment (Fig. 5c). We clarified this in the text "Interestingly, the expression of GSNOR from both, a high Fe resistant variant (GSNOR_Col-0) and a high Fe sensitive variant (GSNOR_Sf-2), was not further induced by high Fe treatment (Fig. 5c), suggesting that the steady-state expression of GSNOR might be key for the increased Fe tolerance" [page 6, line 34ff.].

Question: The tolerance phenotype of overexpression GSNOR in fig 3b is hard to observe as described in line 17-18 page5.

Reply: *This 35S:GSNOR line in Col-0 background (a high-Fe tolerant accession) is only slightly tolerant (yet statistically significant) to 150 μM Fe but not higher concentration of Fe. We have added two more time points between day 3 and day 6 in Figure 3b to clearly show this tolerance pattern in 35s:GSNOR line in the revised manuscript.*

3)NO and H2O2 session;

Question: The authors tried to analyze GSNOR function under the high Fe using the knockout mutant. They showed the NO metabolism and inducible cell death determined by GSNOR function. However, they are poorly distinguished. NO, GSNO and GSH production can be measured.

Reply: *Previous studies have provided good evidence that NO, GSNO and GSH are highly accumulated in the Arabidopsis gsnor knockout mutants (Lee et al, 2008; Chen et al., 2009; Kovacs et al., 2016). Consistently we also found that NO was accumulated in the root meristem of gsnor knockout mutant (Fig 6b). GSNO is a NO-derived molecule, generated by the interaction of NO with reduced glutathione (GSH) (Corpas et al., Front. Plant Sci. 2013). To mechanistically test the roles of NO/GSNO and GSH, we therefore applied NO donor, NO scavenger and GSH inhibitor to roots, and also used other NO accumulation mutants to test the roles of NO and GSH in gsnor mutants sensitive to high Fe, and using these data to reveal the accumulation of NO is responsible for the hypersensitive phenotype in gsnor mutant in response to high Fe (Fig. 6 and Supplementary Fig. 6).*

Question: Additionally, evaluation of cell death and Fe accumulation should provide convincing evidence.

Reply*: We agree with the reviewer that cell death and Fe accumulation* are convincing evidence to test our hypotheses*. We therefore evaluated both: To evaluate cell death, we used both Propidium iodide (PI) and Sytox Orange staining. PI stains the walls of living plant cells but is also used as a marker for loss of membrane integrity and cell death, while Sytox Orange is a cell death marker (Truernit and Haseloff, 2008, Plant Methods). Both PI and Sytox Orange staining were widely used in many studies to detect cell death (Fulcher and Sablowski, 2009, Proc Natl Acad Sci U S A; Hashimura and Ueguchi, 2011, Plant J; Horvath et al., 2017, EMBO J; Hong et al., 2017, Cell). The images of cell death for both wild-type and gsnor mutants are shown in Fig 6d, f and Supplementary Fig. 8. The images in Fig 6d, f may be too small to observe the cell death, therefore we provide higher magnification images in Supplementary Fig. 8. The images in the control condition were also added in the revised Supplementary Fig. 8. To clarify, we*

added in the text "PI stains the walls of living plant cells but is also used as a marker for loss of membrane integrity and cell death, while Sytox Orange is a cell death marker"[page, line 2ff.].
To observe Fe accumulation in the root tip, we used Perls/DAB staining which is one of the most recognized method to detect Fe in vivo and widely used in the field of Fe-related research (Roschzttardtz et al., Plant Physiology. 2009; Reyt et al., Molecular Plant, 2015; Müller et al., Developmental Cell, 2015; Balzergue et al., Nature Communications, 2017). We observed a clear difference of Perls/DAB staining between wild-type and gsnor mutant in the condition of 50 µM Fe (Supplementary Fig. 7a).

Question: Is there any difference of the phenotype related to GSNOR activity among the accessions?
Reply: *Thank you for the comment. We did not assay the phenotype directly related to GSNOR activity such as NO accumulation among the accessions yet, as we think the gsnor mutants suffice for GSNOR function testing in response to high Fe toxicity in the current study.*

Question: Were GSNOR expressed in the root meristem, in which NO accumulation and cell death occurred as the authors mentioned?
Reply: *Thank you for the comment. Yes. The native promoter driven GSNOR-GFP was strongly expressed in the root meristem (Fig. 6c; also see Xu et al., Front. Plant Sci., 2013.*)

Question: Some publications have reported that Fe deficiency and Cd induce NO accumulation including GSNO/GSNOR. How was the high-Fe dependent NO oxidative stress explained?
Reply: *We are also very interested to know how high Fe increases NO-mediated cytotoxicity. NO accumulation could be induced by many conditions including Fe deficiency and Cd stress. The root growth of gsnor mutants has been reported to be resistant to salt stress and copper (Zhou et al., PLOS Genetics, 2016; Peto et al., Plant Cell Rep., 2013). We have also tested the gsnor mutant in response to Fe deficiency, Cd and Cu stresses, but did not find that the root growth of gsnor mutants were more sensitive to these stresses. Therefore, high-Fe dependent NO oxidative stress may be different with that caused by Fe deficiency and other metals. It was reported that Fe could increase the NO decomposition in the presence of H_2O_2 (Farias-Eisner et al., 1996), and also that NO can interact with H_2O_2 to cause a stronger cytotoxic effect through an unknown mechanism as observed in previous studies in plant disease response and cell death in leaves (Delledonne et al., 2001; Lin et al., 2012). Thus, high Fe may intensify the interaction between NO and H_2O_2 to cause a stronger cytotoxic effect, as the gsnor mutants were also very sensitive to H_2O_2 depending the Fe levels (Fig 6f, g and Supplementary Fig 7c,d). While a lot of questions arise when considering the interactions among high Fe, NO and H_2O_2, and while we would like to explore these in the future, this is out of scope of this manuscript.*

Question: Supporting Fig 6c should be test the significance between the WT and the mutant.

Reply: *Thank you for the suggestion. The significant difference between the wild-type and the mutant has been added to the Supplementary Fig. 6 c (now Supplementary Fig. 7c) the revised manuscript.*

Question: Page 8 line 8; how is the difference with "Fe enhance H2O2 damage"

Reply: *Thank you for the improvement. The sentence has been changed to "Hence, similar to nitrosative stress, this result suggests that high Fe enhances H_2O_2 damage in the gsnor mutants [page 8, line24]".*

Question: Page 8 line11; Why were the KO tolerant to paraquat? Are the other toxic metals inducing ROS inhibit the growth of the KO?

Reply: *Paraquat can convert O_2 into the superoxide radical (O_2^{-}) in chloroplasts or the cytoplasm and thereby causes oxidative stress and cell death (Dinis-Oliveira et al., 2008), while Fe reacts with H_2O_2 to generate $OH^{\cdot}$ (a highly active ROS) (Toyokuni, 1996; Galaris and Pantopoulos, 2008). Therefore, one possibility is that NO reacts with O_2^{-} to form the peroxynitrite anion ($ONOO^{-}$) that is less toxic in plants (Delledonne et al., 2001), which may explain the tolerance of the gsnor mutants to paraquat stress. However, the reasons for gsnor mutant tolerance to paraquat are still unresolved (Chen et al., Cell Research, 2009). Unlike the high Fe condition, the root growth of gsnor mutants have been reported to be resistant to salt stress (indirectly inducing in ROS production) and copper (directly involved in ROS production) (Zhou et al., PLOS Genetics, 2016; Peto et al., Plant Cell Rep., 2013).*

4) higher plants session;

Question: The authors mentioned that the aim and result of this study is to help breeding in discussion and background. And also Arabidopsis transgenic line introduced high expression type GSNOR (Col-0 accession) showed the high shoot FW and slightly, but significant, long root length compared to low expression type. Furthermore, the authors used word of "GSNOR confers tolerance". Overexpression line or high expression line/accession should be tested rather than knockout mutants.

Reply: *We thank the reviewer for highlighting this wording issue. While we have shown Arabidopsis that higher expression can confer tolerance, we formally only showed in the other plant species that GSNOR is required for high Fe tolerance. Therefore we have reworded the title of the manuscript to "GSNOR provides plant tolerance to iron toxicity via preventing iron-dependent nitrosative and oxidative cytotoxicity", as well as the section title and the text that relates to the other plant species.*

Question: Supporting fig10e and f were hard to follow.

Reply: *To make it easier to follow, we have added additional wording to it "A subfunctionalization is further supported by the absence of many phenotypes in Ljgsnor1 mutants that are present in Arabidopsis gsnor mutants*[28,29] *that include visible development defects at both young stages (shorter roots under the normal condition) and mature stages (shorter stem, increased branching, reduced fertility and shorter siliques) (Supplementary Fig. 11e and f), as well as by the distinct, moderately anticorrelated (r = 0.2) expression pattern of the two LjGSNOR genes (Supplementary Fig. 12)" [page 10, line 6ff.]*.

5) Discussion

Question: Page11 line 14: GSNOR of human and other species would be compared in supporting fig 9.

Reply: *The comparison of GSNOR sequence and enzyme activity among human, bacterium and plant (including Arabidopsis) has been performed in several previous studies (Liu et al., Nature, 2001; Sakamoto et al., FEBS Lett., 2002; Lee et al., Plant Cell, 2008; Xu et al., Front. Plant Sci., 2013), so we think it is not necessary to repeat this analysis again. Instead, we reference the previous studies in our discussion [page 12, line4ff.].*

Question: I think the discussion would argument from the study of NO metabolism related to GSNOR (GSNO) involved in Fe deficiency and expression/promoter polymorphism explaining variations on the consequence of what authors find.

Reply: *Thank you for the suggestion. We have tested the gsnor mutant in response to Fe deficiency, but did not find the clear difference of root growth between the wild-type and the mutant. Moreover, the expression of Fe deficiency marker genes such as FRO2 and IRT1 was also similar between the wild-type and the mutant when the seedlings transferred from Fe starvation condition to high Fe for 0h, 3h, 6h and 24h (data not shown). Therefore, we do not think that it is worth to discuss the difference of NO metabolism in gsnor mutant between Fe deficiency and Fe toxicity. Secondly, it is a good point to discuss the contribution of the expression/promoter polymorphisms of GSNOR to explain the variations of root tolerance to high Fe and we already have them in the second (line 25 to line 30 of page 10) and third (line 10 to line 14 of page 11) paragraphs in the discussion part.*

Reviewer #3 (Remarks to the Author):

This paper tackles the issue of high iron (Fe) toxicity in plants through screening for genes that confer tolerance to high Fe in Arabidopsis thaliana accessions. Using a GWAS approach, the authors clearly demonstrate that significant variation in Fe tolerance is associated with variant alleles of S-nitrosoglutathione-reductase (GSNOR). This extensive study involves competent, high resolution phenotyping and GWAS statistical analysis, supported by allelic complementation, leaving no doubt that the relevant genetic locus conferring a significant percentage of Fe tolerance has been identified. They go on to provide evidence that GSNOR also confers tolerance to high Fe in rice and Medicago truncatula, expanding the significance to crop plants. The authors state that previous attempts to identify genes involved in tolerance to Fe have not been successful, making their study a landmark in this field and identifying a gene for potential targeted breeding efforts. The actual mechanism by which GSNOR confers Fe tolerance is not illuminated here, although it is linked to the over-accumulation of NO species through studies of Arabidopsis plants that carry a null mutation in GSNOR. Further investigation of the mechanism is clearly outside the scope of the present study.

We thank the reviewer for this assessment of our work and the thorough review.

Question: The authors could better strengthen the significance of the work to agriculture if it were possible to relate the Fe levels used for their studies to those causing Fe toxicity in the field. Statements about Fe toxicity in the field are very general, and overall not particularly informative.
Reply: *Thank you for the suggestions. We added the information about the Fe levels and soil types that could causing Fe toxicity in plants and revised this part in the introduction. "In fact, Fe toxicity represents one of the most widely spread soil constraints for crop production in waterlogged soils, but also in a wide range of soil types including Ferralsols, Acrisols, Fluvisols, Podzols and Gleysols [3], and can cause up to 10% to 90% of yield loss in rice[4,5]. The Fe concentration causing Fe toxicity in rice ranges widely, from 10 to>2000 mg L^{-1} in the soil solution, and is highly dependent on other soil parameters such as geochemistry and nutrient levels, as well as on the particular rice variety[6]" [page 3, line 6 ff.].*

Question: In addition, as shown in Fig. 3b, attempts to increase GSNOR expression conferred only minor change in Fe toxicity. The authors do comment that could be due to the overall high resistance to Fe of the Arabidopsis accession tested. However, it would be more satisfying it the authors actually showed

levels of the GSNOR protein in this and some of their other experiments, which is readily accomplished with available GSNOR antisera. In addition, GSNOR activity can be measured in whole cell extracts.

Reply: *We thank the reviewer for these excellent suggestions. The 35s:GSNOR overexpression used in this study was created and characterized in the previous studies (Achkor et al., Plant Physiol., 2003; Rustérucci et al., Plant Physiol., 2007). It has already been shown that the gene expression and GSNOR activity was increased 8.3 fold and 19 fold in this 35s:GSNOR line respectively (the GSNOR gene overexpression was confirmed for this line in our laboratory), while S-nitrosothiols (SNOs) still remained 80% of the wild-type level (Rustérucci et al., Plant Physiol., 2007). This information has now been added in the second paragraph of discussion in the revision manuscript, "However, there was no tolerance difference at higher levels of Fe (Fig. 3b) suggesting that either the 35S promotor doesn't drive GSNOR expression high enough in the relevant cell types, or there is a limit as to which low NO levels can mediate Fe tolerance, or that in Col-0 sufficiently high levels of GSNOR expression are present and thereby there is no possibility to increase Fe-tolerance further due to feedback mechanisms. The latter is supported by the characterization of the 35s:GSNOR line in which it was determined that while the GSNOR activity was increased 19-fold, the S-nitrosothiols (SNOs) still remained at 80% of the wild-type level[43] [page 11, line 4ff.]"*

Also, to illustrate the effect of the increase of tolerance better, in the revised manuscript we added two more time points to Fig 3b that clearly show the tolerance pattern in 35s:GSNOR line at 150 µM Fe .

Overall, the authors have presented an excellent study that is a roadmap for using GWAS to discover novel genes associated with specific phenotypes.

Additional issues that should be addressed are listed below:

Question: 1) The authors may wish to indicate at first introduction, the accession background of the hot5-2 and hot5-4 mutants, as this is relevant to their subsequent introduction of the A and J haplotype alleles into hot5-4.

Reply: *Thanks for pointing out this issue. It has been added in the revised manuscript.*

Question: 2) Fig. 3b. The authors should either correct the scale of the hot5-2 graph to correspond to the scale of the other graphs, or express all values as a percentage of the growth of the control (50 uM fe?).

Reply: *Thank you for the suggestion. It has been modified as suggested by the reviewer. In this figure, we also added two more time points between day 3 and day 6 to clearly show this tolerance pattern in 35s:GSNOR line in the revision manuscript (Fig. 3b).*

Question: 3) As mentioned above - Fig. 5c. It would be useful to have tested the level of GSNOR protein in these lines. This may provide insight as to whether OE of GSNOR is indeed occurring. Activity of GSNOR can also be tested in seedlings. This is particularly relevant to their discussion, Page 10, lines 276 to 282 and to the possibility of engineering tolerance using this enzyme.

Reply: *We have determined the overexpression of the GSNOR gene and the causality of the alleles to conferring a higher resistance. While it is an excellent suggestion to evaluate enzymatic activity and the molecular regulatory role of these GSNOR variants or SNPs on GSNOR protein level and activity, a careful analysis is needed for this, in particular to accurately account for differences in GSNOR protein content that could otherwise occlude activity differences. We hope to address that carefully in a follow-up project which we think our findings strongly warrants.*

Question: 4) Please state more specifically in the text what the inhibitory mechanism of BSO.

Reply: *Thanks for the suggestion. We have added the sentence "BSO, an inhibitor of gamma-glutamylcysteine synthetase, consequently reducing GSH synthesis" in the revised manuscript [page 7, line 19].*

Question: 5) Fig. 6e. The bottom of the X-axis legend is partially covered by the panel g.

Reply: *Thank you for pointing the issue. It has been corrected in the revised manuscript.*

Question: 6) Supp. Fig. 7a. It is not clear why the authors have a red arrowhead in the wt picture at 50 uM Fe, but not in the mutant picture. I believe the goal here is to show more accumulation in the mutant, but this single picture makes that difficult to confirm.

Reply: *Thank you for pointing the issue. Two frames in the red dot line have been added to the wild-type and gsnor mutant to indicate the difference of Fe staining in Supplementary Fig. 7a in the revised manuscript.*

Question:7) Supp. Fig. 7d. The indication of H2O2 levels has extra "0"s that need to be removed.

Reply: *Thank you for pointing the issue. It has been corrected in the revised manuscript.*

Question:8) Supp. Fig. 10. The Ljgsnor1-1 picture presented for Day 7 and day 17 appear identical. I believe the authors have made an error in constructing this figure.

Reply: *Thank you for paying attention to these images. The seedlings of Ljgsnor1-1 indeed looks very similar between Day 7 and Day 17 (they are the same seedling but pictures on day 7 and day 17*

respectively). Despite 10 days of difference they look almost identical because their growth was almost completely inhibited by 350 µM Fe treatment. However, there are distinct, observable differences as the color of the stem is different (Day 7 seedlings with light green, while Day17 seedings with red) and so is the color of the root tip. In fact, they were on the same plate with Ljgsnor1-2 seedlings which had clear bigger leaves. In order to avoid confusing, we shortened the dotted line between Ljgsnor1-2 and Ljgsnor1-2 so that it is easier to see that the different genotypes are growing on the same plate.

Question:9) Page 9. Line 256 "that present in Arabidopsis gsnor mutants" should be "that are present in Arabidopsis gsnor mutants". This result would also be better explained if the authors added more specifics as to the phenotypes to which they are referring - that is increased branching and reduced fertility.

Reply: *Thanks for the correction. This sentence has been modified to "A subfunctionalization is further supported by the absence of many phenotypes in Ljgsnor1 mutants that are present in Arabidopsis gsnor mutants[28,29] that include visible development defects at both young stages (shorter roots under the normal condition) and mature stages (shorter stem, increased branching, reduced fertility and shorter siliques) (Supplementary Fig. 11e and f), as well as by the distinct, moderately anticorrelated (r = 0.2) expression pattern of the two LjGSNOR genes (Supplementary Fig. 12)" [page 10, line 6 ff.].*

Question:10) Concerning subfunctionalization of the putative two GSNOR genes in Lotus, it should be possible for the authors comment on whether or not these two genes are similarly expressed. They might also consider that stating more specifically that it could be differences in substrate specificity of GSNOR for substrates other than GSNO. Can the authors also specifically state that both genes are in the ADHIII clade, not the ADHI clade?

Reply: *Thank you for the suggestions. According to the information in LOTUS base (https://lotus.au.dk/), both LjGSNOR1 and LjGSNOR2 are predicted to belong to ADHIII clade (this information has been added to the revised manuscript [page 9, line 32]). LjGSNOR1 is strongly and constitutively expressed, while LjGSNOR2 is much less expressed but could be induced by certain conditions such as nodule incubation. However, we do not know the substrate specificity of GSNOR in Lotus japonicus, which is another aspect that would be worthwhile to be investigated further in a follow-up project. We commented on the expression now: "A subfunctionalization is further supported by the absence of many phenotypes in Ljgsnor1 mutants that are present in Arabidopsis gsnor mutants[28,29] that include visible development defects at both young stages (shorter roots under the normal condition) and mature stages (shorter stem, increased branching, reduced fertility and shorter siliques) (Supplementary Fig. 11e and f), as well as by*

the distinct, moderately anticorrelated (r = 0.2) expression pattern of the two LjGSNOR genes (Supplementary Fig. 12) [page 10, line 6 ff.].”

Other questions: Grammatical or other issues:

1) Page 3. Line 55 – “sensitive of primary” should be “sensitivity of the primary”

2) Page 5. Line 108 “and no root” should be “and showed no root”.

3) Page 5. Line 110 “while only 20% inhibition in” should be “while only 20% inhibition was observed in”.

4) Page 9. Line 238 “retarded much more pronounced” should be “retarded much more”

5) Page 9. Line 225 “of Ljgsnor1 mutants at the visible development defects” – seems it should be “in Ljgsnor1 mutants of visible development defects”

6) Page 11. Line 304. “It is widely accepted that Fe toxicity highly dues to generate hydroxyl radical via the Fenton reaction with H2O2”. This sentence makes no sense.

7) Page 11. Line 308. “prevents from cell death” should be “prevents cell death”

8) Page 11. Line 309. “to generating” should be “for generating” or “to generate”

9) Page 11. Line 312. RNS is introduced for the first time with no definition.

10) Page 11. Line 315. “reduction that” should be “reduction, which” in order to make this sentence easier to follow.

Reply: *Thank you very much for these corrections. All of them have been corrected as suggested by the reviewer. For the point of (6), this sentence has been modified to “Fe-catalyzed ROS production is thought to be the major reason for Fe toxicity [page 11, line 27]” in the revised manuscript.*

Reviewer #4 (Remarks to the Author):

Genes are known to be differentially expressed by high iron stress. However, a genetic trait explaining tolerance to iron toxicity is not known, even though iron toxicity represents an agricultural problem for rice on waterlogged acidic soils in Africa and Asia. This manuscript proposes that GSNOR gene expression variation can explain tolerance to high iron apparent as decreased root growth inhibition, and authors suggest that decreased NO in the presence of elevated GSNOR and Fe is the cause for reduced oxidative stress in the root meristem.

In a first part of the manuscript the authors describe GWAS studies using in total several hundred natural variation lines and transgenic Arabidopsis plants to identify and confirm the high iron root length and gene expression phenotype. In a second part the authors investigated the cause of the phenotype using physiological experiments with different transgenic lines, grown in the presence or absence of iron and NO to study the connection of high iron, GSNOR expression and root length. Finally, the authors show in transgenic rice and Lotus loss-of-function plants that low GSNOR expression under high iron is also associated with reduced root growth in other species.

Overall, the findings are interesting and novel in the context of root development and iron and fit to the currents efforts to identify genetic variation traits to high iron or more particularly to root growth inhibition. However, regarding the discussion and importance of the results in terms of agricultural high iron tolerance in Arabidopsis and in crops, major questions remain and here more clear data and analyses are needed to back up the claims that authors want to make in this context.

Major comments:

Question: 1) To claim that GSNOR gene expression contributes to high iron toxicity tolerance, authors should investigate plant development in general, leaf bronzing, a typical symptom of iron toxicity, flowering phenotypes, seed production, in Arabidopsis and also in rice. It remains unclear what is the effect of the root growth trait on general growth and yield of the plants (this also accounts for the introduction, which does not comprise major adaptive effects known to confer high iron tolerance). All analyzed phenotypes were restricted to root growth, especially in the early plant developmental stages. Plants might have adapted in other ways to high iron and the GSNOR effect might only be relevant transiently for some root growth adaptation but may not contribute to overall plant tolerance.

Reply: *Thank you for the suggestions. As root growth is frequently used to evaluate heavy metal tolerance (Wilkins, 1978, New Phytol.) and also for high Fe toxicity in plants (Li et al., J. Exp. Bot., 2015; Reyt et al., Molecular Plant, 2015), we used the root growth as the primary index to evaluate the symptom of iron toxicity. However, and much like the reviewer suggests it is important to look beyond these root growth traits and observe whether phenotypes occur in the shoot. In the case of gsnor knockout mutants, both of roots and true leaves could not grow when Fe concentration was increased from 50 µM (the control) to 350 µM (Fe concentration used for the GWAS screening). The leaves of gsnor knockout mutants were still much smaller than the wild-type at 250 µM Fe (Fig. 3a). To clarify, we now added to the results: "The observed sensitivity to high Fe was not restricted to root growth but encompassed traits in the whole seedling as the leaves of gsnor knockout mutants were much smaller than the wild-type at 250 µM Fe (Fig. 3a)." [page 5, line 23ff.]*

A similar phenotype was also observed in the transgenic lines transformed with two natural GSNOR variants (high Fe tolerant and sensitive respectively). In the results this is stated: "The high Fe root growth tolerance conferred by the GSNOR_Col-0 alleles also correlated with an approximately two-fold increase in shoot biomass under high Fe compared to the sensitive allele that we measured in the T3 lines (Fig. 5b), demonstrating the relevance of GSNOR dependent high Fe tolerance at the organismal level" [page 6, line 28ff.].
In rice, the reduction of height in Osgsnor knockout lines was around 12% more than that in the wild-type when treated with high Fe for 2 weeks (Supplementary Fig. 11a, c). To clarify this effect, we added:" The reduction of height in Osgsnor knockout lines compared to wild-type amounted to 12% when treated with high Fe for 2 weeks. Thus, OsGSNOR contributes to both, root and plant tolerance to Fe toxicity in rice." [page 9, line 22ff.].
In the case of Lotus, leaf growth of both Ljgsnor1-1 and Ljgsnor1-2 homozygous seedlings were much smaller than the wild-type and heterozygous seedlings when grown at 350 μM Fe for 17 days (Supplementary Fig. 11d). To clarify this, we added: "This increased root growth sensitivity was accompanied by a decreased size of the shoot system and increased accumulation of red pigments over time (Supplementary Fig. 11d)." [page 10, line 3ff.]
Taken together, our results support that the relevance of GSNOR dependent high Fe tolerance is not limited to root growth, but at the overall organismal level, and we hope we could point this out with the changes to the manuscript we have made.

Question: 2) In the same line, there are some studies conducted with high iron transcriptome changes and tolerance in rice. The authors should check and discuss whether GSNOR gene expression variation has been found in any of these studies.
Reply: *Thank you for the suggestion. We have checked two transcriptomic data sets in rice in response to high Fe (Quinet et al., Plant Cell & Environ., 2012; Bashir et al., Rice, 2014), but did not find that the GSNOR gene was significantly changed by high Fe. In fact, the expression of GSNOR was also not significantly affected by high Fe treatment in our study (Fig. 5c). We discussed this now: "Interestingly, GSNOR expression is not induced by high Fe itself neither in Arabidopsis (Fig. 5c) nor in rice[41,42], indicating that the base-line expression of GSNOR is important for its relevance for high Fe tolerance." [page 10, line 31ff.].*

Question: 3) Physiological experiments are rather narrow and merely address iron toxicity at the root apex leading to different root lengths of primary roots. Other aspects of NO and iron effects are not experimentally addressed and not even discussed. For example, it has been shown in several studies that

NO has a positive effect on ethylene, that both NO and ethylene promote iron uptake via regulation of transcription factors, which is a response to NO along the root and in the root hair zone. Furthermore, NO can affect regulation of ethylene synthesis and perhaps other components relevant for iron uptake regulation via nitrosylation. No studies are presented to investigate the effect of GSNOR expression on Fe acquisition regulation in roots at the molecular level.

Reply: The topics that the reviewer raises are really interesting and comprehensively highlight the complexity of root growth modulatory pathways and mechanisms. We therefore now made it clear in the discussion how interesting these issues are: *"GSNOR seems to directly participate in the regulation of Fe-induced redox-dependent cytotoxicity. Fe-catalyzed ROS production is thought to be the major reason for Fe toxicity[14-16]. However, studies in yeast and plant indicate that Fe-catalyzed ROS production may not account for Fe toxicity[20,22], which suggests that other components (additional to ROS) might be also required for Fe-mediated toxicity. We demonstrated that GSNOR protects root meristem growth and prevents cell death caused by Fe-dependent NO-induced nitrosative and H_2O_2-induced oxidative toxicity in Arabidopsis. These results revealed that NO is also required to generate Fe-dependent redox toxicity, which advances our understanding of the toxic mechanisms of Fe. Additionally, NO-mediated potassium homeostasis can also participate in the inhibition on the root growth by high Fe[45]. Thus, as the interplay of high Fe, ROS and reactive nitrogen species (RNS) is highly complex[46,47], it will be very interesting to investigate these interactions and their impact of cytotoxicity." [page 11, line 26ff.]. Other than discussing this, we think that any additional experiments are beyond the scope of our study.*

Question: 4) In this context, it would be interesting to know how iron uptake itself is actually affected and what are the iron contents of plants in different organs. Principally, one might assume that short root growth is an advantage in the presence of high metals rather than longer roots, which would have a higher surface for toxic metal uptake. Thus, it seems important for iron tolerance to consider mechanisms that restrict iron uptake into the root cells and restrict long-distance transport of iron to shoots and seeds. So what is the actual effect of GSNOR expression here?

Reply: *These are interesting thoughts. If Fe uptake and transport are the primary factor causing the hyper-sensitive phenotype in gsnor mutants, it is reasonable to assume that short root growth may acquire less Fe and show more tolerance to high Fe than the longer roots. However, RNA-seq and qPCR results (data not shown) suggest that the Fe uptake and transport are not be the primary factor responsible for the high Fe hyper-sensitive phenotype in gsnor mutant (no canonical iron deficiency or transport genes were differently expressed between gsnor mutant and the wild-type). As we responded to Question 1 of this reviewer, our data showed GSNOR is not only required for root tolerance to high Fe,*

but also for leaf growth tolerance to high Fe Arabidopsis, Lotus and rice (Fig. 3a; Fig. 5b; Supplementary Fig. 11a, c, d), thus, the relevance of GSNOR dependent high Fe tolerance is at the overall organismal level. Therefore, we think the sensitivity to high Fe in gsnor mutants is related to Fe-mediated reactive oxygen species (ROS) production or scavenging rather than Fe uptake and transport. It would be interested to investigate how GSNOR-dependent NO signaling or S-nitrosylation regulation integrates Fe and ROS to cause cytotoxicity in the follow-up project.

Question: 5) The authors make the point that high Fe causes nitrosative cytotoxic stress. This raises the question whether it is not possible to prove such effects and detect the effective compounds in plant cells.

Reply: *Thank you for the comment. According to the definition of nitrosative stress in this field, nitrosative stress is the result of the amount of reactive nitrogen species (RNS) exceeding the capacity of the antioxidant machinery, and may induce irreversible damages in all cellular macromolecules including genomic DNA (Ortega et al., Cancer, 2010). Therefore, the amount of nitric oxide (NO), NO-derived compounds and DNA damage could be used to indicate the level of nitrosative cytotoxic stress (Bai et al., Nitric Oxide, 2012; Moylan et al., Neuroscience and Biobehavioral Reviews, 2014). Our results showed high Fe could increase NO accumulation in the root meristem of wild-type (Fig. 6b) and clearly cause cytotoxic stress (indicated by both propidium iodide and sytox orange staining) in the root meristem of gsnor mutants with high accumulation of NO (Supplementary Figure 8). Therefore, high Fe enhances nitrosative cytotoxic stress.*

Additional comments:

Question: 1) How was it controlled by authors that Fe is soluble at 350 µM under the respective pH? Is Fe taken up and can it be measured that plants differ in Fe contents under normal and high iron?

Reply: *In order to make Fe soluble in the medium, we used Fe(III)EDTA which is soluble in water. 350 µM Fe to 500 µM Fe are widely used as high Fe in previous studies in Arabidopsis (Reyt et al., Molecular Plant, 2015; Li et al., J. Exp. Bot., 2015), while Fe concentrations up to several millimole are still often used to the assay of Fe toxicity in rice (Engel et al., J Plant Nutr Soil Sc., 2012; Wu et al., Rice, 2014). We also observed the inhibition of Fe on the growth of seedlings was increased with Fe concentrations from 50 µM to 500 µM in the growth medium. The leaves started to die under 500 µM Fe condition in several Arabidopsis accessions, which indicating Fe still could be taken up even Fe concentration up to 500 µM. Therefore, we think Fe uptake and transport should be still working under 350 µM Fe condition.*

Question:2) The authors should describe in earlier paragraphs what was exactly the variation, which phenotypes were observed.

Reply: *We have added the description in the first sentence in the Result part as "To identify genetic variants that confer plant tolerance to high Fe, we made use of natural variation of primary root growth responses in Arabidopsis thaliana to high Fe, as primary root growth is frequently used to evaluate heavy metal tolerance[25] and also for high Fe toxicity in plants[22,26]."[page 4, line 6ff.]*

Question:3) Explain better T-and A-alleles in the text.

Reply: *Thank you for the suggestion. A sentence has been added to clarify this: "The T-variant (54%) of this lead SNP was associated with higher Fe tolerance and the A-variant (46%) was associated with lower Fe tolerance within these 319 accessions (Supplementary Fig. 3c)." [page 4, line 27ff.].*

Question:4) Explain earlier in the text how GSNOR expression correlates with the phenotype.

Reply: *The related modification has been added in the second paragraph in the Result part in the revised manuscript. "Indeed we found one gene, AT5G43940, which encodes a S-nitrosoglutathione reductase (GSNOR), which displayed a supporting expression pattern: it was significantly different between T-variant accessions (higher GSNOR expression) and A-variant accessions (lower GSNOR expression) in both roots and shoots under control conditions (Supplementary Fig. 3d and e; Supplementary Table 1)." [page 5, line 4ff.].*

Question:5) Explain better the use of different statistical methods for the physiological assays and correct it in the figure legends.

Reply: *Thank you for the suggestion. We have modified the description for the different statistical methods used for the physiological assays in the Method of the revised manuscript. To clarify, we added "Significant differences between two samples for time-course experiments were determined with Student's t-test. Significant differences for multiple comparisons for single point experiment was determined by one-way or two-way ANOVA with Tukey's HSD test as indicated in figure legends." [page 22, line 18ff.].*

Reviewers' comments:

Reviewer #1 (Remarks to the Author):

This reviewer feels that the mechanistic point is still a weak point of the paper. Given that the authors presented solid genetic evidence revealing a critical agronomic trait, the current version of the MS is perhaps acceptable for publication in Nat Commun.

Minor points

One of the incorrect citations: lines 197-198, nox1 was identified as a mutant with high NO level by Zhen-Ming Pei lab (Science, 2004, 305: 1968). The nox1 mutant is allelic to previously identified cue1. The authors should cite Pei et al. paper, while the citation of the other two papers is optional.

Reviewer #2 (Remarks to the Author):

I understood almost all of the author's responses, which was well and kindly explained. However, I still have some comments to the responses.
-Not only root length but also mechanism of natural variation (direct phenotype) for the function of GSNOR in Fe tolerance should be presented between allele differences (promoter variants). The direct physiological evidence of natural variation via GSNOR is important for the future agriculture mentioned by the authors.
-Even if you use the root length at high Fe at GWAS in this study, please make clear distinction between "root length at high Fe" and "Fe tolerance" defined by the authors across the manuscript.
-How many times and plant did you conduct for the each microscope experiment? Please mention the information in the method or each legend. And the author should prove independent datasets, not only data of one root.

Reviewer #3 (Remarks to the Author):

The authors have adequately addressed the major technical questions about the GWAS analysis and addressed all of the minor points of the reviewers.
There are two issues that I still think remain incompletely addressed:
1) Reviewer Question: The authors could better strengthen the significance of the work to agriculture if it were possible to relate the Fe levels used for their studies to those causing Fe toxicity in the field. Statements about Fe toxicity in the field are very general, and overall not particularly informative.

The authors provide some more quantitative information on the content of Fe in the soil in their introduction, but do not try to relate it to the content in their experiments at any point. They need to bring this issue back into the discussion, especially if they wish to make the claim of increasing Fe tolerance by this manipulation.

2) Reviewer Question: In addition, as shown in Fig. 3b, attempts to increase GSNOR expression conferred only minor change in Fe toxicity. The authors do comment that could be due to the overall high resistance to Fe of the Arabidopsis accession tested. However, it would be more satisfying it the authors actually showed levels of the GSNOR protein in this and some of their other experiments, which is readily accomplished with available GSNOR antisera. In addition, GSNOR activity can be measured in whole cell extracts.

Although in their response the authors state the expression level of the GSNOR OE line was

demonstrated by the individuals providing these lines, it remains for the authors to confirm the lines behave as “advertised” especially after subsequent propagation. This is a simple control – to perform western analysis on roots of this line to demonstrate the extent of increased GSNOR levels. Activity measurements are not much more difficult, but perhaps not necessary. Again, if they are indicating that increasing GSNOR could promote Fe tolerance, they need a better demonstration of this. Figure 3 would certainly not support this as a mechanism for increased Fe tolerance.

As part of their response to this question they also added to the text on page 11: “However, there was no tolerance difference at higher levels of Fe (Fig. 3b) suggesting that either the 35S promotor doesn't drive GSNOR expression high enough in the relevant cell types, or there is a limit as to which low NO levels can mediate Fe tolerance, or that in Col-0 sufficiently high levels of GSNOR expression are present and thereby there is no possibility to increase Fe-tolerance further due to feedback mechanisms.”

This sentence is convoluted and difficult to follow. The authors need to reconsider explaining their point.

Other:
I would like to see a supplemental figure comparing the GSNOR amino acid sequences from rice lotus and Arabidopsis.

Supplemental Fig. 8a. Legend refers to red triangle, but the authors have changed the figure and removed the red triangle and added red lines. The legend needs to be changed correspondingly.

Reviewer #4 (Remarks to the Author):

The authors have addressed several comments, but still two comments are left-over, and the data should be carefully collected and evaluated.

1) Several reports indicate that NO promotes iron uptake. The authors say in their rebuttal letter that “RNA-seq and qPCR results (data not shown) suggest that the Fe uptake and transport are not be the primary factor responsible for the high Fe hyper-sensitive phenotype in gsnor mutant (no canonical iron deficiency or transport genes were differently expressed between gsnor mutant and the wild-type)”. This is a very interesting result and can be discussed as it supports the ideas of the authors. The authors should therefore show the data in the manuscript since their discussion is obviously based on these gene expression data and include these conclusions in their discussion.

2) This point also relates to comments that other reviewers have made. The authors often refer to other publications that have shown certain effects at biochemical level, gene or protein abundance for example in the gsnor mutant or in response to high Fe and NO. Despite of that, it remains very critical to prove that the reported amounts of molecules are indeed present in the growth conditions and in the plants that the authors have used here in this manuscript. These conditions are certainly different from what is published, and it is control phenotypes. I would therefore strongly recommend that authors control their experimental systems by checking GsNOR activity, protein abundance and cytotoxic compounds.

We would like to thank the four reviewers very much for the time, effort, thought and constructive comments on our manuscript. We have considered the comments, concerns and suggestions and revised the manuscript to address these. Please find our point-by-point responses below. The paragraphs in regular font are the comments from reviewers, and the paragraphs in italic are our responses. We also highlighted edits in the revised manuscript with red color. We believe our revisions further improved the quality of our manuscript and hope that the reviewers agree and accept our responses and modifications.

Reviewers' comments:

Reviewer #1 (Remarks to the Author):

This reviewer feels that the mechanistic point is still a weak point of the paper. Given that the authors presented solid genetic evidence revealing a critical agronomic trait, the current version of the MS is perhaps acceptable for publication in Nat Commun.

We thank the reviewer for accepting our responses and recommendation for publication.

Minor points

Question: One of the incorrect citations: lines 197-198, nox1 was identified as a mutant with high NO level by Zhen-Ming Pei lab (Science, 2004, 305: 1968). The nox1 mutant is allelic to previously identified cue1. The authors should cite Pei et al. paper, while the citation of the other two papers is optional.

Reply: *Thanks for correcting this citation. This paper (He et al., Science, 2004, 305: 1968) has been added as* ***Reference NO.36*** *into the revised references.*

Reviewer #2 (Remarks to the Author):

I understood almost all of the author’s responses, which was well and kindly explained. However, I still have some comments to the responses.

We thank the reviewer for taking the time to review our responses and explanations.

Question: -Not only root length but also mechanism of natural variation (direct phenotype) for the function of GSNOR in Fe tolerance should be presented between allele differences (promoter variants). The direct physiological evidence of natural variation via GSNOR is important for the future agriculture mentioned by the authors.

Reply: *As shown in Figure 5a-c, we have demonstrated that the two GSNOR variants from high Fe tolerance or sensitive accessions caused the significant difference of GSNOR expression levels, which leads to the root growth phenotype. We assumed that the change of GSNOR transcript can lead to the different accumulation of GSNOR protein and function but we hadn't tested this. In the revised manuscript, we therefore took another step and showed via GSNOR western blots that the previously observed difference at the transcript level between these two GSNOR variants indeed results in the different accumulation of GSNOR protein (Figure 5d). We therefore now show that the GSNOR variants cause expression level differences, which are in turn translated into different GSNOR protein levels.*

Question: -Even if you use the root length at high Fe at GWAS in this study, please make clear distinction between "root length at high Fe" and "Fe tolerance" defined by the authors across the manuscript.

Reply: *Thanks for the comment. Fe tolerance has been defined as the ratio of root length under high Fe and root length under the control conditions in the section of Genome-wide Association Studies (GWAS) in the Method [page20, line9], and also in the first paragraph in the Result part [page4. Line 9].*

Question: -How many times and plant did you conduct for the each microscope experiment? Please mention the information in the method or each legend. And the author should prove independent datasets, not only data of one root.

Reply: *Thank you for the suggestion. For microscopy observations, at least 8 individual roots were analyzed for each genotype in a given condition in each independent experiment. At least 2 independent experiments were performed. The representative images from one experiment were presented. These sentences have been added to the Microscopy section of in Method in the revised manuscript[page 25, line 14 ff.]. The number of plants in Fig. 6d ,f have been already noted in Figure legends of Fig. 6e, g.*

Reviewer #3 (Remarks to the Author):

The authors have adequately addressed the major technical questions about the GWAS analysis and addressed all of the minor points of the reviewers.

There are two issues that I still think remain incompletely addressed:

1) **Reviewer Question**: The authors could better strengthen the significance of the work to agriculture if it were possible to relate the Fe levels used for their studies to those causing Fe toxicity in the field. Statements about Fe toxicity in the field are very general, and overall not particularly informative.

The authors provide some more quantitative information on the content of Fe in the soil in their introduction, but do not try to relate it to the content in their experiments at any point. They need to bring this issue back into the discussion, especially if they wish to make the claim of increasing Fe tolerance by this manipulation.

Reply: *Thanks very much for the suggestion again. As mentioned in the introduction[page3, line8ff.], the Fe concentration causing Fe toxicity in rice ranged from 10 to 2000 mg.L-1 (about 179 µM to 36 mM) in soil solutions. The Fe concentration used in this study ranged from 150 µM to 1000 µM (350 µM for GWAS screening and most experiments, 150 µM for Arabidopsis gsnor mutant analysis, 1000 µM for rice gsnor mutant assay, the control medium MS and ½ MS containing 100 µM or 50 µM Fe). All of these concentrations were very close to the lower range of Fe toxicity that had been described in rice. This means that the Fe concentration used in this study could be available in the field. However, Fe toxicity in plant not only depends on the Fe concentration, but also is highly dependent on other soil parameters such as geochemistry and nutrient levels, as well as on the particular rice variety. For example, non-toxic Fe concentrations can cause a very strong Fe-dependent inhibition on the root growth under low phosphate conditions (Müller et al., 2015, Dev. Cell 33, 216). The particular Fe concentration leading to Fe toxicity also is species dependent: Wild type rice grew quite normal (only 8 % reduction in the root length) in 1000 µM Fe (Figure 7c), while many Arabidopsis natural accessions showed a very strong*

inhibition (even death) when 500 µM Fe was provided in our pilot GWAS screening. Therefore, we think that that it is very difficult to directly compare between the Fe levels used in studies with those causing Fe toxicity in the field. Instead it would better to directly test gsnor mutant and GSNOR natural variants in rice and lotus in real soils that have Fe toxicity problem in future. We therefore added to the discussion "For this additional studies need to be conducted in the relevant soils as Fe toxicity in plant not only depends on the Fe concentration in the soil, but is highly dependent on other soil parameters such as geochemistry and nutrient levels and on the particular genetic background.[page 10, line 24ff.]"

2) **Reviewer Question**: In addition, as shown in Fig. 3b, attempts to increase GSNOR expression conferred only minor change in Fe toxicity. The authors do comment that could be due to the overall high resistance to Fe of the Arabidopsis accession tested. However, it would be more satisfying it the authors actually showed levels of the GSNOR protein in this and some of their other experiments, which is readily accomplished with available GSNOR antisera. In addition, GSNOR activity can be measured in whole cell extracts.

Although in their response the authors state the expression level of the GSNOR OE line was demonstrated by the individuals providing these lines, it remains for the authors to confirm the lines behave as "advertised" especially after subsequent propagation. This is a simple control – to perform western analysis on roots of this line to demonstrate the extent of increased GSNOR levels. Activity measurements are not much more difficult, but perhaps not necessary. Again, if they are indicating that increasing GSNOR could promote Fe tolerance, they need a better demonstration of this. Figure 3 would certainly not support this as a mechanism for increased Fe tolerance.

As part of their response to this question they also added to the text on page 11: "However, there was no tolerance difference at higher levels of Fe (Fig. 3b) suggesting that either the 35S promotor doesn't drive GSNOR expression high enough in the relevant cell types, or there is a limit as to which low NO levels can mediate Fe tolerance, or that in Col-0 sufficiently high levels of GSNOR expression are present and thereby there is no possibility to increase Fe-tolerance further due to feedback mechanisms."

This sentence is convoluted and difficult to follow. The authors need to reconsider explaining their point.

Reply: *We thank the reviewer for these excellent suggestions. As suggested by the reviewer, we performed the experiments of GSNOR western blot and GSNOR enzyme activity in both 35S:GSNOR line and GSNOR variant complemental lines. These results have been added into the revision manuscript*

(Figure 5d and Supplementary Figure 14). Similar to previous results (Rustérucci et al., 2007, Plant Physiol. **143**, 1282*), both GSNOR protein and enzyme activity were highly increased in the 35S:GSNOR line compared to the wild type (Supplementary Figure 14). In GSNOR variant complemented lines, the accumulation of GSNOR protein was significantly higher in GSNOR_Col-0 (#1-2 and #3-3) than that in GSNOR_Sf-2 (#4-4 and #9-4) (Figure 5d), the same holds true for the GSNOR activity (Supplementary Fig. 6c). This is similar to the GSNOR expression pattern between GSNOR_Col-0 and GSNOR_Sf-2 lines (Figure 5c). Taken together, the gene expression difference between GSNOR_Col-0 and GSNOR_Sf-2 variants or 35S: GSNOR line leads to differences at GSNOR protein level and GSNOR activity.*

As suggested by the reviewer, we also have modified the related discussion in the revision manuscript. It reads now: "As in this line, the 35S promotor leads to a very high expression of GSNOR protein, as well as GSNOR activity[43] (Supplementary Figure 14), our results suggest that improved tolerance to Fe toxicity requires an optimal level of GSNOR (such as a level close to the GSNOR_Col-0 variant in Ws-4 accession background) or a high GSNOR expression level in a specific cell type rather than in all cell types as conferred by the 35S promoter." [page11, line5ff.].

Other:

Question: I would like to see a supplemental figure comparing the GSNOR amino acid sequences from rice lotus and Arabidopsis.

Reply: *The comparison of GSNOR amino acid sequences among rice, lotus and Arabidopsis had already been included in supplemental figure 10c.*

Question: Supplemental Fig. 8a. Legend refers to red triangle, but the authors have changed the figure and removed the red triangle and added red lines. The legend needs to be changed correspondingly.

Reply: *Thanks for pointing out this issue. The corresponding legend has been changed into "The area surrounded by red dash lines indicates the area of differential Fe accumulation in the root tips of wild-type and hot5-2" in the revised manuscript.*

Reviewer #4 (Remarks to the Author):

The authors have addressed several comments, but still two comments are left-over, and the data should

be carefully collected and evaluated.

1) Several reports indicate that NO promotes iron uptake. The authors say in their rebuttal letter that "RNA-seq and qPCR results (data not shown) suggest that the Fe uptake and transport are not be the primary factor responsible for the high Fe hyper-sensitive phenotype in gsnor mutant (no canonical iron deficiency or transport genes were differently expressed between gsnor mutant and the wild-type)". This is a very interesting result and can be discussed as it supports the ideas of the authors. The authors should therefore show the data in the manuscript since their discussion is obviously based on these gene expression data and include these conclusions in their discussion.

Reply: *As pointed out by the reviewer, several reports indicate that NO promotes iron uptake. However, this effect was only from iron deficiency conditions (Graziano and Lamattina, 2007; Chen et al., 2010), while NO almost has no effect on either the expression of the Fe-acquisition genes or the ferric reductase activity at high level of Fe (García et al., Plant Physiol Biochem. 2011, 49:537). However, we are hesitant to include the RNAseq data as we don't think it would do this rich and complex data justice to be buried in the supplement of this manuscript, we'd rather use it as central part of a subsequent study. However, we see the point of this reviewer and have included qPCR data that leads to the same conclusions. In particular, we checked the expression of bHLH100, bHLH39, FIT and FER1 (the major gene of ferritin proteins that binding and storing Fe in plant) in the roots of gsnor mutant and the wild-type in response to high Fe treatment. All of these genes were expressed at a similar level as in WT. This is the text we added to the discussion [page 12, line 6ff.]:*
"Several reports indicate that NO promotes Fe uptake under Fe deficiency conditions[48,49], while NO almost has no effect on either the expression of the Fe-acquisition genes or the ferric reductase activity at high level of Fe[50]. Since it has been reported that under control conditions three Fe deficiency responsive genes (bHLH100, 2.7-fold; bHLH039, 2.0-fold; bHLH038, 1.6-fold) were upregulated in the shoots of gsnor mutant compared to wild-type [29], we explored the possibility that accumulation of NO in the roots of the gsnor mutant might increase Fe transport and accumulation in high Fe conditions. We therefore measured the root expression of bHLH100, bHLH39, FIT1 all of which are key iron deficiency induced transcription factors that directly activate the expression of ferric-chelate reductase FRO2 and high-affinity ferrous iron transporter IRT1 to increase Fe uptake and accumulation[51]. Expression of none of these genes was different between gsnor mutant and the wild-type in response to high Fe treatment (Supplementary Figure 15). The same held true for expression of FER1, which encodes for the major ferritin protein that bind and store Fe in plants (Supplementary Figure 15). This strongly suggests that Fe uptake and transport are not among the major factors for the high Fe susceptibility of the gsnor mutant."

2) This point also relates to comments that other reviewers have made. The authors often refer to other publications that have shown certain effects at biochemical level, gene or protein abundance for example in the gsnor mutant or in response to high Fe and NO. Despite of that, it remains very critical to prove that the reported amounts of molecules are indeed present in the growth conditions and in the plants that the authors have used here in this manuscript. These conditions are certainly different from what is published, and it is control phenotypes. I would therefore strongly recommend that authors control their experimental systems by checking GsNOR activity, protein abundance and cytotoxic compounds.

Reply: *As recommended by this reviewer,* we now *performed the experiments of GSNOR western blot and GSNOR enzyme activity in the 35S:GSNOR line as well as in GSNOR variant complemented lines, and . These results have been added into the revision manuscript (Fig. 5d, Supplementary Fig. 6c and Fig.14). Similar to previous reports (Rustérucci et al., 2007, Plant Physiol.* **143***,* 1282*), both GSNOR protein and enzyme activity were highly increased in the 35S:GSNOR line compared to the wild type (Supplementary Figure 14). To confirm NO accumulation in gsnor mutant, DAF-FM staining had been already used to show the high accumulation of NO as shown in Fig. 6b. Therefore, 35S:GSNOR line and gsnor mutants used in this study were indeed comparable to the previous reports.*

REVIEWERS' COMMENTS:

Reviewer #2 (Remarks to the Author):

The author well described for the comments. Current version of MS would be acceptable for publication in Nature Communication.

Reviewer #3 (Remarks to the Author):

THe authors have now adequately addressed all the reviewer comments and better aligned their conclusions with the data presented.

Reviewer #4 (Remarks to the Author):

My comments have been addressed, and I have no more questions at this point.
Thank you for highlighting the changes.

We would like to thank for all reviewer’s recommendations for publication on our manuscript.

REVIEWERS' COMMENTS:

Reviewer #2 (Remarks to the Author):

The author well described for the comments. Current version of MS would be acceptable for publication in Nature Communication.

Reviewer #3 (Remarks to the Author):

THe authors have now adequately addressed all the reviewer comments and better aligned their conclusions with the data presented.

Reviewer #4 (Remarks to the Author):

My comments have been addressed, and I have no more questions at this point.
Thank you for highlighting the changes.
